# Growth and Stress Tolerance Comprise Independent Metabolic Strategies Critical for Staphylococcus aureus Infection

**DOI:** 10.1128/mBio.00814-21

**Published:** 2021-06-08

**Authors:** Gyu-Lee Kim, Thomas A. Hooven, Javiera Norambuena, Barry Li, Jeffrey M. Boyd, Jason H. Yang, Dane Parker

**Affiliations:** a Department of Pathology, Immunology and Laboratory Medicine, Rutgers New Jersey Medical School, Newark, New Jersey, USA; b Center for Immunity and Inflammation, Rutgers New Jersey Medical School, Newark, New Jersey, USA; c Richard King Mellon Institute for Pediatric Research, University of Pittsburgh Medical Center, Pittsburgh, Pennsylvania, USA; d Department of Pediatrics, University of Pittsburgh School of Medicine, Pittsburgh, Pennsylvania, USA; e Department of Biochemistry and Microbiology, Rutgers, The State University of New Jersey, New Brunswick, New Jersey, USA; f Department of Microbiology, Biochemistry and Molecular Genetics, Rutgers New Jersey Medical School, Newark, New Jersey, USA; g Center for Emerging and Re-emerging Pathogens, Rutgers New Jersey Medical School, Newark, New Jersey, USA; New York University School of Medicine

**Keywords:** *Staphylococcus aureus*, host-pathogen interactions, metabolism, pathogenesis, pneumonia

## Abstract

Staphylococcus aureus is an important pathogen that leads to high morbidity and mortality. Although S. aureus produces many factors important for pathogenesis, few have been validated as playing a role in the pathogenesis of S. aureus pneumonia. To gain a better understanding of the genetic elements required for S. aureus pathogenesis in the airway, we performed an unbiased genome-wide transposon sequencing (Tn-seq) screen in a model of acute murine pneumonia. We identified 136 genes important for bacterial survival during infection, with a high proportion involved in metabolic processes. Phenotyping 80 individual deletion mutants through diverse *in vitro* and *in vivo* assays demonstrated that metabolism is linked to several processes, which include biofilm formation, growth, and resistance to host stressors. We further validated the importance of 23 mutations in pneumonia. Multivariate and principal-component analyses identified two key metabolic mechanisms enabling infection in the airway, growth (e.g., the ability to replicate and form biofilms) and resistance to host stresses. As deep validation of these hypotheses, we investigated the role of pyruvate carboxylase, which was important across multiple infection models and confirmed a connection between growth and resistance to host cell killing. Pathogenesis is conventionally understood in terms of the host-pathogen interactions that enable a pathogen to neutralize a host’s immune response. We demonstrate with the important bacterial pathogen S. aureus that microbial metabolism influences key traits important for *in vivo* infection, independent from host immunomodulation.

## INTRODUCTION

Staphylococcus aureus is a major human pathogen that causes significant morbidity and mortality (1). Methicillin-resistant S. aureus (MRSA) is now prevalent across the globe and is not limited to acquisition in nosocomial settings. Community-acquired MRSA (CA-MRSA) strains are typically more virulent (2) and cause multiple types of infection, including pneumonia, skin and soft tissue infections, osteomyelitis, and bacteremia (3). Pneumonia caused by the CA-MRSA strain USA300 is widespread across the United States (3, 4) and can affect young infants and the elderly, as well as healthy individuals (5); an effective vaccine has yet to be developed. The full repertoire of genetic elements required for pathogenesis is still not fully understood.

Systematic identification of the factors required for S. aureus infection is throughput limited in conventional mouse models of infection. However, advances in forward genetics and sequencing technologies have facilitated the use of transposon sequencing (Tn-seq), a robust technique combining highly saturated transposon mutant libraries with massively parallel sequencing, to determine fitness under different *in vitro* and *in vivo* conditions (6). Tn-seq has been used to characterize gene functions across entire bacterial genomes (7) and to identify virulence factors contributing to survival during infection (8). In the context of S. aureus infection, studies have examined the genetic requirements during skin and bone infection (9–11), highlighting the power of this technique to identify the genetic requirements during host infection. Recently, a transposon library was used in S. aureus USA300 to define the genes required for nitric oxide (NO) resistance (9). While much is known about the role of factors in systemic infection, far less is known about the genetic requirements for pulmonary infection with S. aureus.

In this study, we performed a transposon sequencing (Tn-seq) screen in the context of acute murine pneumonia to identify new genetic elements required for S. aureus pathogenesis in the airway. Using the CA-MRSA strain USA300, Tn-seq identified a large number of genes important for survival in the airway. Strains with transposon insertions in metabolic genes were significantly depleted. We individually screened a large population of transposon mutants to identify numerous new mutations associated with bacterial growth, oxygen consumption, biofilm formation, virulence factor production, and resistance to macrophage killing and antimicrobial compounds. Strains with mutations in metabolism were heavily selected against, and their role in infection was confirmed *in vivo*. Genes involved in central metabolism had a significant correlation between extracellular and intracellular survival rate, and extracellular acidification rate (ECAR) with intracellular survival, while these associations could be uncoupled with other metabolic pathways. This was confirmed through principal-component analysis that showed the majority of variance in the data could be attributed to two major elements, growth and stress tolerance. We validated our findings genetically, focusing on pyruvate carboxylase, which was identified across multiple studies and was confirmed here to be important across all infection models studied. While most dogma is that bacteria produce factors to protect themselves against the host response, we identify and verify that bacterial metabolism influences several elements of pathogenesis that aid *in vivo* survival. We define the contribution of a multitude of genes for S. aureus pneumonia and highlight the critical nature of bacterial metabolism in persistence and host defense.

## RESULTS

### Identification of genes important for S. aureus pneumonia using Tn-seq.

**FIG 1:**
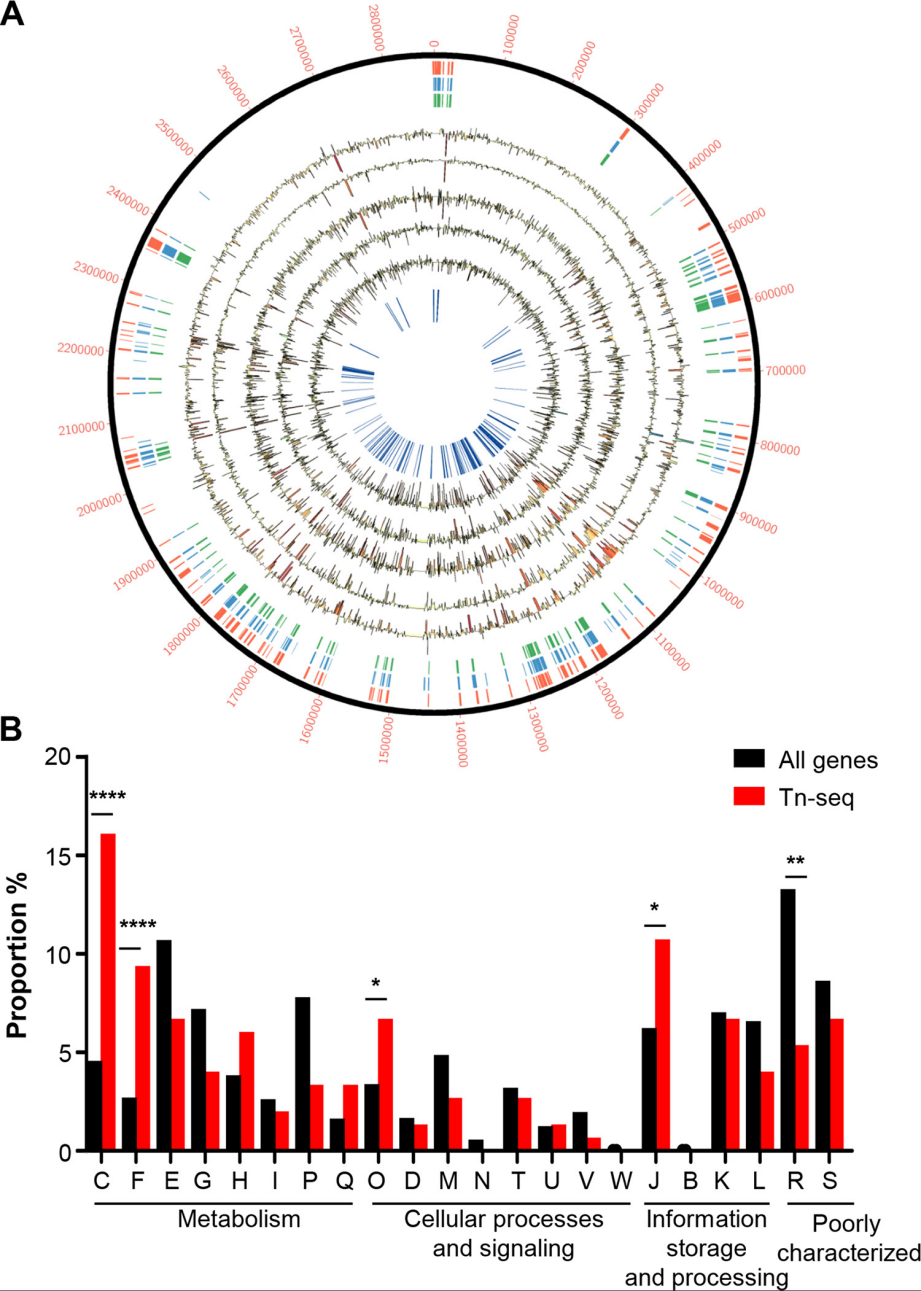
S. aureus genes required for pulmonary infection identified by transposon sequencing (Tn-seq) analysis. (A) Circos plot of Tn-seq data. The outer ring provides the base count of the S. aureus genome. The red tiles mark genes designated essential from this study. The following ring with blue tiles depicts genes called essential in prior studies, and the green tiles are genes classed across all studies as essential. Five rings (black) depict read counts for each mouse analyzed. Mouse reads are shown as histograms with decreases (yellow to red) and increases (green to blue) shaded underneath based on the magnitude of change. The innermost ring (blue) highlights genes identified as conditionally essential in the pneumonia infection model. (B) Genes identified by Tn-seq were classed into clusters of orthologous groups (COGs) and their proportion was compared to the full genome. C, energy production and conversion; F, nucleotide transport and metabolism; E, amino acid transport and metabolism; G, carbohydrate transport and metabolism; H, coenzyme transport and metabolism; I, lipid transport and metabolism; P, inorganic ion transport and metabolism; Q, secondary metabolite biosynthesis, transport, and catabolism; O, posttranslational modification, protein turnover, and chaperones; D, cell cycle control, cell division, and chromosome partitioning; M, cell wall/membrane/envelope biogenesis; N, cell motility; T, signal transduction; U, intracellular trafficking, secretion, vesicular transport; V, defense mechanisms; W, extracellular structures; J, translation, ribosomal structure, and biogenesis; B, chromatin structure and dynamics; K, transcription; L, replication, recombination, and repair; R, general function prediction; S, unknown function. ****, *P* < 0.0001; **, *P* < 0.01; and *, *P* < 0.05, compared to the whole genome.

To identify novel factors involved in pulmonary pathogenesis, we conducted an unbiased genome-wide screen using Tn-seq. We conducted this screen in our model of acute pneumonia using a Tn-seq library in the MRSA strain USA300 LAC, a leading cause of disease in the United States (9). Initial analysis identified 272 genes essential for S. aureus viability (Fig. 1A, red tiles; see also Table S2a in the supplemental material), which are independently supported by prior studies (Fig. 1A, blue and green tiles) (9–11). Mice were infected for 24 h with the transposon library, and sequencing reads were compared to our input. Of the remaining 2,341 genes, our analysis identified 136 conditionally essential genes (Table S2b) that are important for S. aureus infection in the airway (Fig. 1A, inner blue lines). These include mutations in *purA*, *purB*, and *clpX*, which have been shown to be important in various models of infection (12–14). Clusters of orthologous groups (COG) analysis (15) of these genes revealed significant enrichment in processes associated with energy production (16.1% versus 4.6%; *P* < 0.0001) and nucleotide transport and metabolism (9.4% versus 2.5%; *P* < 0.0001) (Fig. 1B). Consistent with the enrichment in energy and metabolism, a high proportion of our Tn-seq hits (41%) were already curated in Kyoto Encyclopedia of Genes and Genomes (KEGG) metabolic pathways (16); a high proportion of the conditionally essential genes were part of the tricarboxylic acid (TCA) cycle. These findings suggested that metabolic processes play a critical role in S. aureus pneumonia.

10.1128/mBio.00814-21.10 TABLE S2 (A) Essential genes identified by transcriptome sequencing (Tn-seq) and (B) conditionally essential genes identified by Tn-seq. Download Table S2, XLSX file, 0.04 MB. Copyright © 2021 Kim et al. 2021 Kim et al. https://creativecommons.org/licenses/by/4.0/ This content is distributed under the terms of the Creative Commons Attribution 4.0 International license.

### *In vitro* validation of identified metabolic genes as important for macrophage defense, hemolytic activity, and growth.

We validated the results from this Tn-seq experiment using several *in vitro* assays designed to probe the virulence and fitness phenotypes associated with these conditionally essential genes. From the 136 genes identified from our analysis, 80 were available as single-transposon-insertion mutants in the S. aureus USA300 Nebraska transposon mutant library (NTML) (7, 8); we utilized these 80 mutants for our subsequent validation experiments.

**FIG 2:**
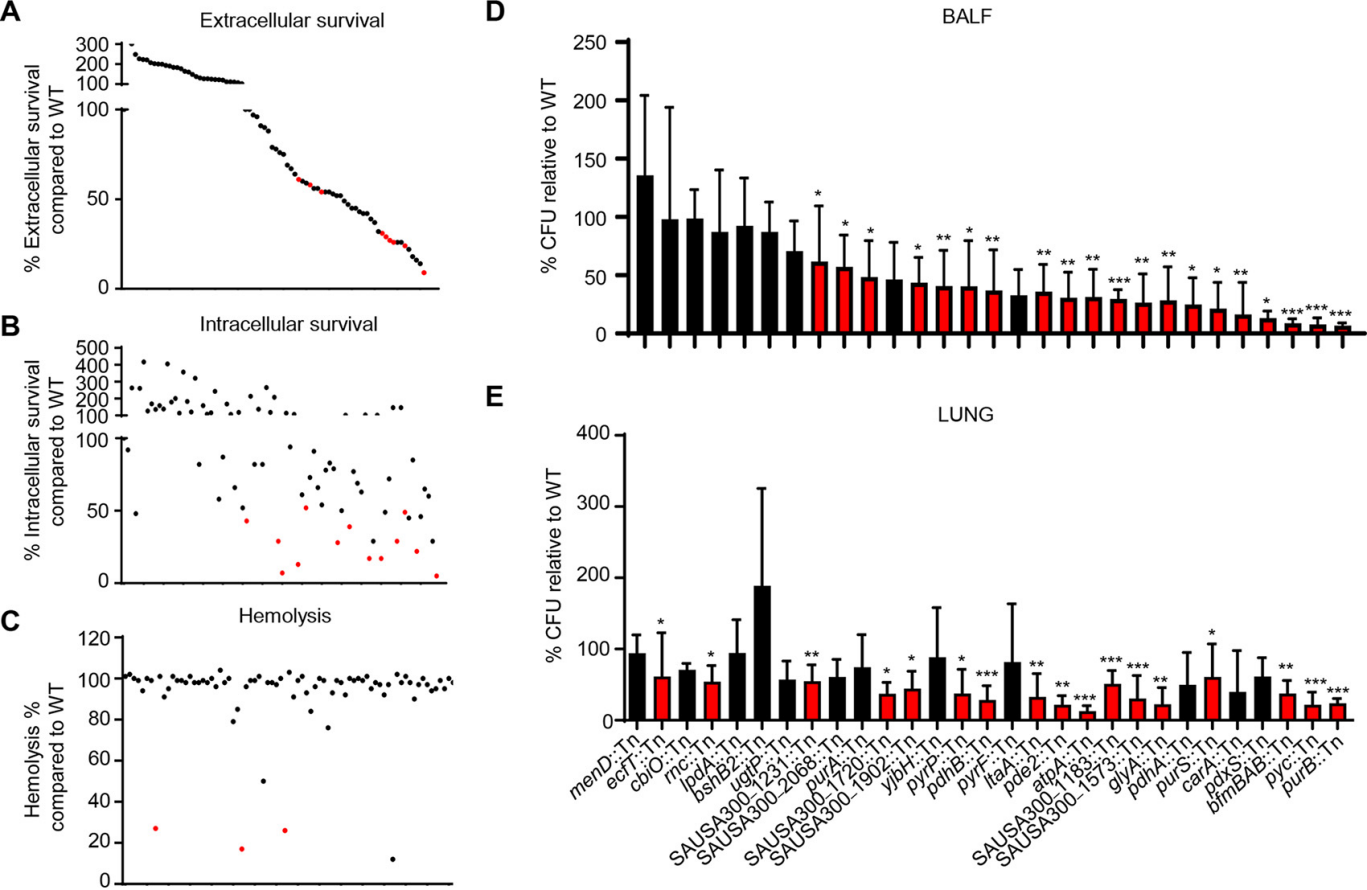
*In vitro* and *in vivo* validation of transposon insertion mutants reveal critical roles for metabolism in host cell survival, hemolytic activity, and pathogenesis of S. aureus. Genes identified as conditionally essential were screened using single-insertion transposon mutants from the S. aureus NTML library. (A) Extracellular killing assay with murine macrophages 2 h after infection and (B) intracellular gentamicin protection assay 4 h after infection. (C) Hemolysis assay on spent culture supernatants. Each dot represents a mutant. Data are arranged based on extracellular data. Red dots highlight mutants with significant changes (*, *P* < 0.05). Each dot represents a mutant. *n* = 4 from two independent experiments with data showing the mean value. Mice were intranasally infected with 10^7^ CFU of wild-type (WT) S. aureus, and selected mutants and bacterial counts were quantified 24 h later. Bacterial counts in (D) bronchoalveolar lavage fluid (BALF) and (E) lung tissue. Graphs show mean with standard deviation. Red bars and dots highlight mutants with significant changes (*P* < 0.05). ***, *P* < 0.001; **, *P* < 0.01; *, *P* < 0.05. *N* = 8.

To better understand how these conditionally essential genes mediate host-pathogen interactions, we assessed the susceptibility of strains to host cell killing using a mouse macrophage survival assay (Fig. 2A and B). Coculture of these 80 mutants with RAW264.7 macrophages revealed 22 mutants that were significantly more susceptible to extracellular killing and 18 mutants that had decreased intracellular survival (>50% decrease compared to the wild type [WT]) (Table 1). While some mutants were defective in both survival assays, defects in intracellular survival were observed in the absence of reduced extracellular survival. While these differences could be due to the capacity of the strains to adhere to the macrophages, we did independently quantify changes in cell viability and phagocytosis for these 18 mutants to control for potential artifacts and did not observe any significant difference in viability or phagocytic uptake in comparison to that of WT cells (see Fig. S1 in the supplemental material). These experiments revealed 5 gene mutants with decreases (and statistical significance) in both extracellular and intracellular survival (*aroA2*, *pyrF*, *scpB*, *clpC*, and *pdhA*), suggesting that those genes might contribute to the persistence in host cells during infection.

**TABLE 1:** Screening of mutants identified by Tn-seq analysis^*b*^

Gene identifier^*a*^	Gene name	Results for:													
		Intracellular survival assay		Extracellular survival assay		Hemolysis assay		Temperature screening at:^*d*^			Protein A^*e*^	Bacterial count^*f*^			
												BALF^*h*^		Lung	
		Mean % of WT ± SD	*P*	Mean % of WT ± SD	*P*	Mean % ± SD	*P*	RT^*g*^	45°C	37°C		Mean (CFU/ml) ± SD	*P*	Mean (CFU/ml) ± SD	*P*
WT		100 ± 27		100 ± 24		101 ± 2		+	+	+	+	12,937 ± 22,057		770,621 ± 1,472,623	
SAUSA300_1127	*smc*	121 ± 38		160 ± 33	**	98 ± 4		+	+	+	+				
SAUSA300_0017	*purA*	7 ± 4	***	78 ± 22		99 ± 1		+	+	+	+	1,271 ± 640	*	192,500 ± 135,620	
SAUSA300_1889	*purB*	13 ± 8	***	67 ± 8		96 ± 8		+	+	+	+	1,288 ± 549	***	144,250 ± 60,327	***
SAUSA300_0965	*folD*	73 ± 22		60 ± 33		76 ± 31		+	+	+	+				
SAUSA300_0995	*pdhC*	94 ± 40		75 ± 49		93 ± 7		+	+	+	+				
SAUSA300_2079	*fba*	87 ± 8		122 ± 47		79 ± 29		+	+	+	+				
SAUSA300_1126	*rnc*	82 ± 38		138 ± 95		98 ± 6		−	−	+	+	4,586 ± 1,874		332,500 ± 148,107	*
SAUSA300_0918	*ugtP*	29 ± 14		14 ± 8		101 ± 3		+	+	+	+	4,150 ± 1945		301,000 ± 260,178	
SAUSA300_1193	*glpD*	116 ± 62		127 ± 41		104 ± 1		+	+	+	+				
SAUSA300_0994	*pdhB*	320 ± 216	*	148 ± 72		99 ± 2		+	+	+	+	4,014 ± 2,778	**	177,143 ± 107,968	***
SAUSA300_2439	*galU*	69 ± 41		47 ± 18		97 ± 4		+	+	+	+				
SAUSA300_1902		66 ± 30		111 ± 37		96 ± 3		+	+	+	+	2,275 ± 1,242	*	212,500 ± 113,610	*
SAUSA300_0870	*rexA*	49 ± 16		37 ± 27		98 ± 3		+	+	+	+				
SAUSA300_1720		29 ± 15	*	79 ± 35		91 ± 10		+	+	+	+	3,325 ± 1926		136,500 ± 96,586	*
SAUSA300_1465	*bfmBAB*	48 ± 58		248 ± 179		99 ± 4		−	+	+	+	450 ± 193	***	185,000 ± 89,283	**
SAUSA300_1573		91 ± 64		59 ± 17		93 ± 10		−	+	+	+/−	3,000 ± 1,555	**	180,000 ± 90,185	***
SAUSA300_0489	*ftsH*	114 ± 68		76 ± 16		101 ± 1		+	+	+	+				
SAUSA300_2037	*cshA*	29 ± 9		42 ± 20		102 ± 3		+	+	+	+				
SAUSA300_1682	*ccpA*	79 ± 48		54 ± 28		99 ± 0		+	+	+	+				
SAUSA300_2060^*c*^	*atpA*	17 ± 11	*	43 ± 21		12 ± 12		+	+	+	+	1,575 ± 1,341	**	54,250 ± 47,225	***
SAUSA300_1590	*relA*	108 ± 73		127 ± 88		96 ± 2		−	+	+	+				
SAUSA300_1650	*pde2*	113 ± 85		182 ± 43		98 ± 2		+	+	+	+	1,529 ± 1,000	**	104,857 ± 62,095	**
SAUSA300_1474		82 ± 30		96 ± 34		97 ± 5		+	+	+	+				
SAUSA300_2058	*atpD*	158 ± 58		200 ± 61		91 ± 8		−	+	+	+				
SAUSA300_1246	*acnA*	260 ± 115	**	226 ± 134	*	94 ± 14		+	+	+	+				
SAUSA300_2003	*rimIispAispA*	60 ± 35		16 ± 11		100 ± 3		−	+	+	+				
SAUSA300_1098	*pyrE*	54 ± 27		56 ± 22		98 ± 6		+	+	+	+				
SAUSA300_1518	*cshB*	72 ± 21		32 ± 10		90 ± 8		+	+	+	+				
SAUSA300_2064	*atpB*	82 ± 39		100 ± 75		98 ± 1		+	+	+	+				
SAUSA300_1683	*aroA2*	22 ± 8	**	24 ± 14	*	95 ± 8		+	+	+	+				
SAUSA300_1090		39 ± 11	*	52 ± 17		95 ± 1		+	+	+	+				
SAUSA300_1183		43 ± 25	*	102 ± 29		101 ± 0		−	+	+	+	1,586 ± 422	***	225,714 ± 89,230	***
SAUSA300_2174	*ecfT*	78 ± 49		56 ± 22		92 ± 4		+	+	+	+	5,675 ± 5,018		322,500 ± 336,102	*
SAUSA300_1016	*cyoE*	52 ± 45		107 ± 48		99 ± 6		+	+	+	+				
SAUSA300_0539	*ilvE*	28 ± 19	**	54 ± 11		100 ± 2		−	+	+	+				
SAUSA300_0869	*rexB*	66 ± 15		58 ± 13	*	99 ± 2		+	+	+	+				
SAUSA300_1097	*pyrF*	29 ± 9	***	29 ± 9	****	100 ± 2		+	+	+	+	3,843 ± 3,983		402,857 ± 510,496	
SAUSA300_2646	*trmE*	83 ± 32		54 ± 35	**	100 ± 10		+	+	+	+				
SAUSA300_1444	*scpB*	49 ± 11	**	26 ± 9	****	94 ± 6		−	+	+	+				
SAUSA300_0510	*clpC*	52 ± 34	**	61 ± 16	*	99 ± 3		−	+	+	+				
SAUSA300_0552	*bshB2*	118 ± 64		110 ± 58		99 ± 7		+	+	+	+	6,088 ± 2,314		762,500 ± 567,746	
SAUSA300_0969	*purS*	136 ± 43		202 ± 29		101 ± 3		+	+	+	+	6,229 ± 7,314	*	412,500 ± 347,224	*
SAUSA300_1432		85 ± 21		26 ± 14		99 ± 7		+	+	+	+				
SAUSA300_1356	*aroB*	102 ± 27		42 ± 15		98 ± 3		+	+	+	+				
SAUSA300_0387	*pbuX*	102 ± 43		52 ± 35		101 ± 2		+	+	+	+				
SAUSA300_0961	*qoxC*	263 ± 58	****	300 ± 153	***	100 ± 3		+	+	+	+				
SAUSA300_1470	*ispA*	17 ± 8	*	39 ± 5		101 ± 5		−	−	+	+				
SAUSA300_0917	*itaA*	265 ± 218		91 ± 60		100 ± 1		+	+	+	+	1,843 ± 1,324	**	108,575 ± 131,318	**
SAUSA300_1138	*sucC*	168 ± 69		120 ± 43		85 ± 4		+	+	+	+				
SAUSA300_2068		201 ± 127		183 ± 92		99 ± 11		−	+	+	+	3,645 ± 2,216	*	269,091 ± 139,818	
SAUSA300_0945	*menF*	77 ± 31		49 ± 18		98 ± 3		+	+	+	+				
SAUSA300_0903	*yjbH*	179 ± 68		190 ± 62		99 ± 2		+	+	+	+/−	1,675 ± 995	**	249,750 ± 147,231	
SAUSA300_0364	*ychF*	357 ± 130	*	177 ± 91		101 ± 3		+	+	+	+				
SAUSA300_2073	*tdk*	50 ± 15		53 ± 38		99 ± 2		+	+	+	+				
SAUSA300_1014	*pyc*	58 ± 2		123 ± 56		100 ± 1		+	+	+	+	2,675 ± 1,136	***	325,000 ± 208,601	***
SAUSA300_0959	*fmtA*	46 ± 9		22 ± 9		100 ± 2		+	+	+	+				
SAUSA300_1112	*stp1*	65 ± 22		18 ± 9		98 ± 3		+	+	+	+				
SAUSA300_0946	*menD*	169 ± 100		207 ± 105	*	27 ± 23	***	+	+	+	+	6,614 ± 3,591		514,286 ± 153,499	
SAUSA300_0958		127 ± 22		220 ± 31	*	99 ± 4		+	+	+	+				
SAUSA300_1015	*ctaA*	405 ± 80	****	193 ± 54		101 ± 1		+	+	+	+				
SAUSA300_1640	*icd*	106 ± 91		69 ± 40		84 ± 23		+	+	+	+				
SAUSA300_1641	*gltA*	183 ± 127		164 ± 51		98 ± 3		+	+	+	+				
SAUSA300_2067	*glyA*	137 ± 106		97 ± 56		98 ± 7		+	+	+	+	3,250 ± 1,309	**	267,500 ± 159,262	**
SAUSA300_1231		92 ± 44		352 ± 227	***	102 ± 2		+	+	+	+	3,450 ± 2,282	*	287,500 ± 143,403	**
SAUSA300_1167	*pnpA*	158 ± 45		131 ± 64		100 ± 1		+	+	+	+				
SAUSA300_1255	*fmtC*	61 ± 33		64 ± 40		100 ± 1		+	+	+	+				
SAUSA300_2175	*cbiO*	103 ± 69		45 ± 20		97 ± 3		+	+	+	+	6,100 ± 2,125		390,000 ± 106,904	
SAUSA300_1569		138 ± 63		199 ± 112		95 ± 11		+	+	+	+				
SAUSA300_1248		243 ± 91		125 ± 17		98 ± 9		+	+	+	+				
SAUSA300_1570		417 ± 229	****	222 ± 131	**	100 ± 4		+	+	+	+				
SAUSA300_1173		63 ± 46		45 ± 27		92 ± 8		+	+	+	+				
SAUSA300_2065	*mnaA*	45 ± 13		26 ± 14		95 ± 3		+	+	+	+				
SAUSA300_2645	*gidA*	208 ± 95		88 ± 48		103 ± 1		+	+	+	+				
SAUSA300_1095	*carA*	146 ± 79	****	31 ± 14	*	97 ± 1		+	+	+	+	2,183 ± 1,716	**	231,000 ± 173,647	
SAUSA300_1096	*carB*	147 ± 101		27 ± 20	*	97 ± 2		+	+	+	+				
SAUSA300_0504	*pdxS*	106 ± 69		111 ± 94		17 ± 14	****	+	+	+	+	3,063 ± 1,344	*	337,500 ± 153,971	
SAUSA300_1092	*pyrP*	118 ± 44		90 ± 33		26 ± 22	***	+	+	+	+	2,714 ± 1,476	*	206,857 ± 191,231	*
SAUSA300_0993	*pdhA*	5 ± 3	*	9 ± 3	**	92 ± 4		−	−	+	+	775 ± 623	*	140,750 ± 93,771	
SAUSA300_0996^*c*^	*lpdA*	214 ± 164		100 ± 93		50 ± 20		−	−	+	−	6,243 ± 3,436		422,857 ± 128,804	

a We could not confirm the SAUSA300_2071 strain, although it is listed in the NTML.

b Significance was as follows: *, *P* < 0.05; **, *P* < 0.01; ***, *P* < 0.001; ***, *P* < 0.0001.

c Strains with mutations in these genes had significantly reduced hemolytic activity but also reduced overnight growth, which is factored into these values.

d +, Growth noted; −, no growth or very limited noted.

e Detection of protein A was represented as follows: +, strong expression; +/−, weak expression; −, no detection (see Fig. S5 in the supplemental material).

f Bacterial count from the pneumonia model shown in Fig. 2.

g RT, room temperature.

h BALF, bronchoalveolar lavage fluid.

10.1128/mBio.00814-21.1 FIG S1 Cell viability and phagocytosis of strains with reduced intracellular persistence. (A) Macrophages were infected with wild-type (WT) Staphylococcus aureus and mutants that showed significant decreases in the intracellular gentamicin protection assay. Cells were collected after a 2-h incubation (multiplicity of infection [MOI] = 1), and viable cells were counted following trypan blue staining. All values (*N* = 4) provided are from two independent experiments. Bars represent means with standard deviation. (B) Murine macrophages were infected with fluorescently labeled (AF647) S. aureus mutants, and cells were collected 5 min or 30 min after incubation and detected by flow cytometry. All values (*N* = 4) provided are from two independent experiments. Bars represent standard deviation of the means. ****, *P* < 0.0001; ***, *P* < 0.001; *, *P* < 0.05. Download FIG S1, TIF file, 0.4 MB. Copyright © 2021 Kim et al. 2021 Kim et al. https://creativecommons.org/licenses/by/4.0/ This content is distributed under the terms of the Creative Commons Attribution 4.0 International license.

To further assess the physiology of these S. aureus mutants, we quantified their hemolytic activity, protein A expression, and growth at different temperatures. S. aureus secretes a multitude of toxins that can lyse host cells, including red blood cells (5, 17). We screened our collection of 80 mutants through a quantitative hemolysis assay and identified three mutants (*menD*::Tn, *pdxS*::Tn, and *pyrP*::Tn) that were significantly decreased in hemolytic activity (Fig. 2C), including *menD*, which was previously identified (18). *atpA* and *lpdA* mutants also exhibited reduced hemolytic activity but also had decreased growth (see Fig. S2 in the supplemental material). Interestingly, while mutation in one of the major toxins, alpha-toxin, led to a 30% to 40% decrease in red cell lysis, these five mutations exhibited >70% decreases in hemolysis (Table 1), revealing a critical role of metabolism in S. aureus virulence.

10.1128/mBio.00814-21.2 FIG S2 Mutations with reduced growth. Overnight cultures of S. aureus were diluted 100-fold and grown in Luria-Bertani (LB) broth. Optical density was measured at 1-h intervals. All values (*N* = 3) provided are from three independent experiments. Bars indicate standard deviation. ***, *P* < 0.001. Download FIG S2, TIF file, 0.1 MB. Copyright © 2021 Kim et al. 2021 Kim et al. https://creativecommons.org/licenses/by/4.0/ This content is distributed under the terms of the Creative Commons Attribution 4.0 International license.

Protein A is a major surface protein important for causing infection in the airway (19, 20). We quantified levels of protein A expression for these 80 mutants by Western blot analysis (Table 1; see also Fig. S3 in the supplemental material). The majority of mutations did not have consistently altered expression of protein A, except for mutations in SAUSA300_1573, *yjbH* and *tdk*, with low levels consistently detected. The *lpdA*::Tn strain did not have any detectable protein A (Fig. S3).

10.1128/mBio.00814-21.3 FIG S3 Immunoblots detecting protein A levels in S. aureus mutants compared to that in the WT. The levels of protein A in S. aureus mutants were determined by Western blot analysis. The samples were run on an SDS-PAGE gel and blotted with anti-protein A (42 kDa). Download FIG S3, TIF file, 0.8 MB. Copyright © 2021 Kim et al. 2021 Kim et al. https://creativecommons.org/licenses/by/4.0/ This content is distributed under the terms of the Creative Commons Attribution 4.0 International license.

We also assayed growth under different temperatures for all 80 mutants at 25°C, 37°C, and 45°C (Table 1). Several mutants were identified with defects at low and high temperatures without changes at 37°C, including mutations in *scpB*, *clpC*, and *ilvE* (Table 1). Strains with mutations in *ispA*, *pdhA*, or *lpdA* were sensitive to all temperatures based on plate growth, and by performing growth curves we identified that 5 strains (*pdhA*::Tn, *lpdA*::Tn, *atpA*::Tn, *carB*::Tn, and *pdxS*::Tn) had defective growth rates at 37°C in liquid culture (Fig. S2).

### Strains harboring transposon insertions in metabolic pathways are significantly attenuated *in vivo*.

We further validated our Tn-seq results *in vivo* by infecting mice with individual transposon mutants in our acute pneumonia model. Based on our *in vitro* validation experiments, we selected 29 transposon insertion mutants, including top hits from the Tn-seq analysis and other select genes for *in vivo* validation. We individually infected mice with each of these 29 mutants in two independent experiments and verified that 23 mutant strains were significantly attenuated in acute pneumonia (Fig. 2D and E). Many of these strains were defective in the TCA cycle, ATP production, or purine and pyrimidine metabolism (Table 1). We also observed that the two mutants (SAUSA300_1573::Tn and *yjbH*::Tn) included with reduced protein A were also attenuated. Within this set of mutants, six genes encoding hypothetical proteins were identified as important for survival in the pneumonia model (Table 1). Collectively, these *in vitro* and *in vivo* data support a critical role for S. aureus metabolism in acute pneumonia.

### Metabolism contributes to properties important for infection.

**FIG 3:**
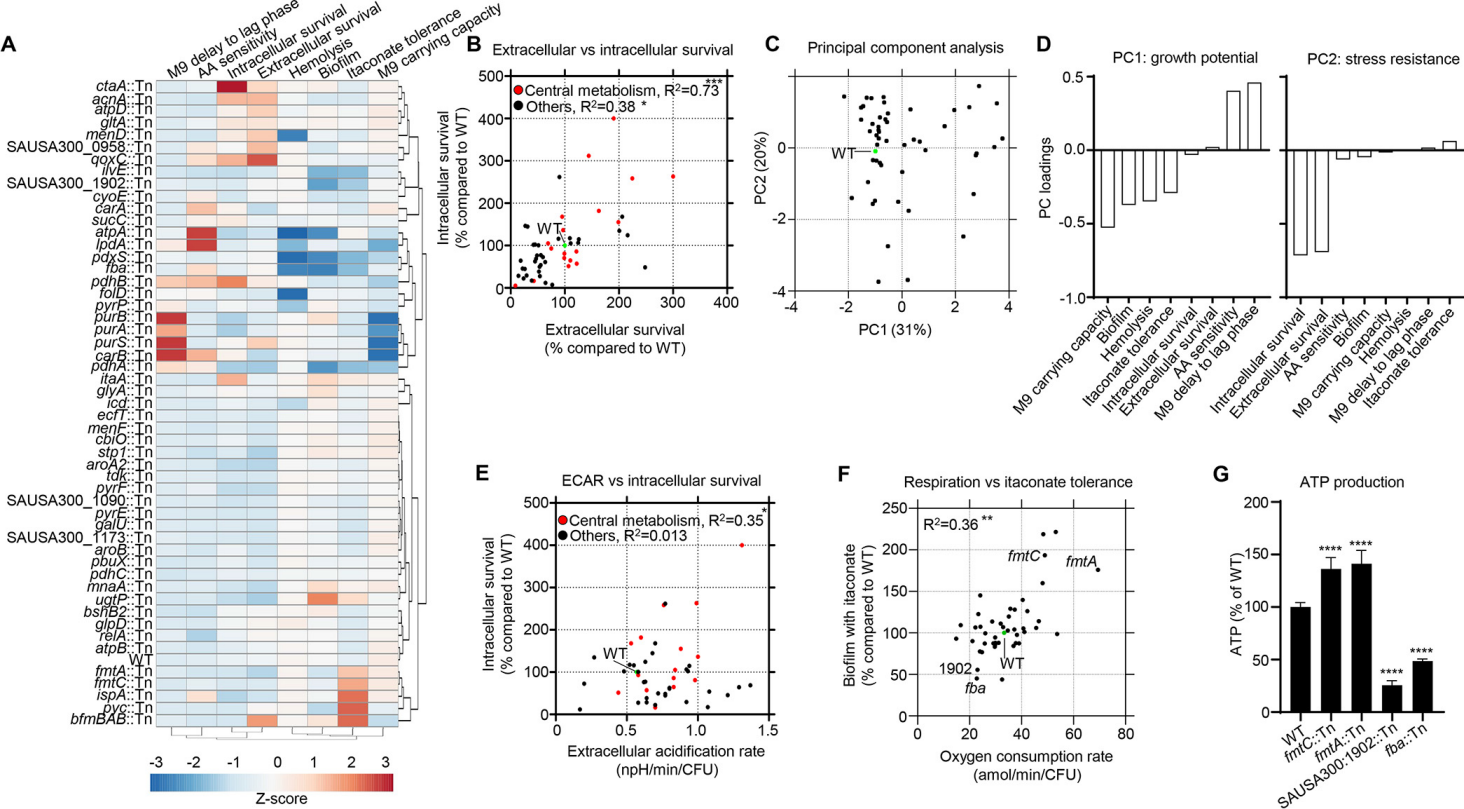
Growth and resistance to host stress are major factors in attenuation of metabolic mutants. (A) Hierarchical clustering heatmap summarizing phenotypic data for metabolically related mutations. (B) Correlation plot of extracellular and intracellular macrophage survival comparing central metabolism mutants (red) with other mutants (black). The WT is shown for reference (green). (C) Principal-component analysis (PCA) of metabolism gene data. (D) Coefficient plots of principal components 1 and 2 (PC1 and PC2). Correlation plots of (E) extracellular acidification rate (ECAR) with intracellular macrophage survival and (F) biofilm formation with itaconate and oxygen consumption rate. Each point represents a mutant. (G) ATP levels in strains shown relative to that in the WT. *N* = 4. ****, *P* < 0.0001; **, *P* < 0.01; *, *P* < 0.05.

Given the predominance of mutations in metabolic genes (Fig. 1B and Table 1), we focused our efforts on understanding how S. aureus metabolism contributes to pathogenesis with several phenotypic assays. We further examined the capacity of 52 strains with transposon insertions in genes from diverse metabolic pathways to grow in complex (LB) or minimal (M9) growth media. Very few mutant strains demonstrated growth defects in LB (Fig. S2), while several mutants, including those defective in glycolysis or purine biosynthesis, exhibited increased lag phases or decreased carrying capacities (maximum growth supported by medium) in M9 medium (Table 2 and Fig. 3A). These mutants are also likely to exhibit growth defects *in vivo*, especially considering how metabolites present in infected tissue may be depleted during infection (21).

**TABLE 2:** Functional characterization of metabolic mutants^*c*^

Gene ID	Gene name	Pathway^*a*^	Results for:											
			AA out of lag (h)^*b*^	M9 medium		M9 out of lag (h)	% Biofilm		% Itaconate		OCR^*d*^		ECAR^*e*^	
				Max OD (mean ± SD)	*P*		Mean ± SD	*P*	Mean ± SD	*P*	Mean (pmol/min) ± SD	*P*	Mean (mpH/min) ± SD	*P*
Wild type			6.22	1.1 ± 0.03		2.75	100 ± 19.09		100 ± 39.23		33.32 ± 8.79		0.57 ± 0.18	
SAUSA300_2079	*fba*	Glycolysis	11.51	0.84 ± 0.08	****	3.53	27.04 ± 7.24	****	45.51 ± 13.29	****	22.74 ± 7.37	***	0.83 ± 0.47	**
SAUSA300_0993	*pdhA*	“	10	0.51 ± 0.12	****	10	35.33 ± 5.26	****	48.36 ± 7.94	***	NA		NA	
SAUSA300_0994	*pdhB*	“	13.4	0.56 ± 0.02	****	11.21	75.42 ± 9.72		89.49 ± 35.07		NA		NA	
SAUSA300_0995	*pdhC*	“	5.66	0.98 ± 0.04		1.88	97.5 ± 27.89		111.21 ± 15		31.98 ± 9.28		0.58 ± 0.03	
SAUSA300_0996	*lpdA*	“	NA	0.38 ± 0.3	****	7.64	89.61 ± 23.13		72.90 ± 18.45	*	NA		NA	
SAUSA300_2067	*glyA*	“	7.17	0.94 ± 0.01	*	3.43	121.67 ± 31.52		105.84 ± 14.60		45.61 ± 22.26		1 ± 0.38	****
SAUSA300_1138	*sucC*	TCA	9.34	0.91 ± 0.03		3.29	96.75 ± 19.34		106.66 ± 39.93		22.22 ± 0.98	*	0.53 ± 0.10	
SAUSA300_1640	*icd*	“	5.75	1.06 ± 0.03	****	3.25	109.25 ± 35.93		105.37 ± 40.85		40.67 ± 8.63		0.84 ± 0.29	**
SAUSA300_1641	*gltA*	“	7.5	1.09 ± 0.03	**	3.23	108.5 ± 24.61		119.63 ± 25.29		35.07 ± 6.38		0.6 ± 0.21	
SAUSA300_1246	*acnA*	“	4.8	1.02 ± 0.04		2.51	77.08 ± 34.94	*	85.63 ± 20.98		29.82 ± 9.97		0.76 ± 0.31	*
SAUSA300_1014	*pyc*	“	5.54	0.83 ± 0.04	****	2.85	66.53 ± 18.67	****	145.21 ± 36.90	*	24.09 ± 6.32	***	0.64 ± 0.32	
SAUSA300_1902		PPP	5.98	0.94 ± 0.12	***	3.84	40.58 ± 11.60	****	55.61 ± 21.59	***	23 ± 3.24	*	0.83 ± 0.13	**
SAUSA300_0961	*qoxC*	Oxphos	11.25	0.85 ± 0.14	***	2.7	93.17 ± 25.80		99.53 ± 39.15		26 ± 10.86	*	0.99 ± 0.44	**
SAUSA300_1015	*ctaA*	“	7.2	1.04 ± 0.03	****	3.25	108.67 ± 22.10		94.39 ± 31.18		29.83 ± 6.04		1.31 ± 0.59	***
SAUSA300_1016	*cyoE*	“	9.75	0.93 ± 0.07		3.29	71.75 ± 23.55	**	77.57 ± 20.88		23.98 ± 12.66	*	0.44 ± 0.20	
SAUSA300_2058	*atpD*	“	5.06	1.09 ± 0.04		3.21	105.5 ± 24.78		113.55 ± 20.16		28.71 ± 4.86		0.88 ± 0.43	**
SAUSA300_2060	*atpA*	“	NA	0.65 ± 0.08	****	4.37	32.17 ± 7.98	****	128.04 ± 16.49		37.35 ± 11.25		0.7 ± 0.31	
SAUSA300_2064	*atpB*	“	5.13	1.07 ± 0.08		3.24	102.25 ± 18.80		88.08 ± 40.16		31.17 ± 2.58		0.98 ± 0.21	***
SAUSA300_0017	*purA*	Purine	5.72	0.28 ± 0.11	****	13	96.08 ± 22.11		81.07 ± 47.75		NA		NA	
SAUSA300_0969	*purS*	“	5.75	NA	****	NA	95.92 ± 15.84		104.44 ± 44.57		30.25 ± 5.25		0.27 ± 0.09	***
SAUSA300_1590	*relA*	“	3.47	1.02 ± 0.06		3.94	102.17 ± 19.62		92.99 ± 28.19		14.83 ± 1.20	****	0.92 ± 0.37	**
SAUSA300_1889	*purB*	“	5.52	NA	****	NA	121.92 ± 21.09		82.94 ± 25.03		25.66 ± 2.46	*	0.17 ± 0.06	****
SAUSA300_1092	*pyrP*	Pyrimidine	5.8	0.66 ± 0.05	****	7.61	108.58 ± 43.11		98.6 ± 29.03		53.72 ± 3.87	***	0.55 ± 0.05	
SAUSA300_1095	*carA*	“	13.12	1.11 ± 0.07	*	3.16	93.73 ± 34.28		84.35 ± 22.03		36.89 ± 9.49		0.68 ± 0.14	
SAUSA300_1096	*carB*	“	13.71	NA	****	NA	89.59 ± 26.99		103.89 ± 38.06		NA		NA	
SAUSA300_1097	*pyrF*	“	6.89	0.99 ± 0.03		3.25	100 ± 26.40		103.97 ± 19.26		37.24 ± 15.57		0.92 ± 0.49	**
SAUSA300_1098	*pyrE*	“	5.54	0.98 ± 0.01		2.94	92.88 ± 19.33		90.19 ± 24.52		21.12 ± 2.81	***	1.08 ± 0.27	****
SAUSA300_2073	*tdk*	“	6.25	1.01 ± 0.02		3.47	104.08 ± 20.82		107.01 ± 57.41		32.93 ± 7.05		0.72 ± 0.10	*
SAUSA300_1173		Lipid	5.78	1.08 ± 0.06	***	3.6	95.33 ± 37.01		83.18 ± 26.53		29.7 ± 1.56		0.8 ± 0.12	**
SAUSA300_1193	*glpD*	“	5.43	0.98 ± 0.04		2.89	97.33 ± 19.21		87.38 ± 25.61		39.05 ± 7.16		0.94 ± 0.54	**
SAUSA300_0918	*ugtP*	“	5.46	0.93 ± 0.12	*	2.03	161.39 ± 34.77	****	159.81 ± 32.57	****	48.1 ± 10.01	**	0.64 ± 0.17	
SAUSA300_0539	*ilvE*	AA	4.33	0.84 ± 0.03	****	2.03	51.58 ± 10.27	****	43.77 ± 5.70	****	32.5 ± 13.56		0.58 ± 0.27	
SAUSA300_1356	*aroB*	“	5.12	1.06 ± 0.04		3.3	98.5 ± 18.10		87.15 ± 33.93		25.83 ± 3.60	*	0.48 ± 0.14	
SAUSA300_1465	*bfmBAB*	“	5	0.64 ± 0.01	****	3.35	125.67 ± 41.29	**	221.61 ± 75.28	****	53.18 ± 20.27	**	0.77 ± 0.15	**
SAUSA300_1683	*aroA2*	“	5.62	1.02 ± 0.02		3.23	95.5 ± 15.95		97.66 ± 43.35		38.21 ± 9.71		0.7 ± 0.26	
SAUSA300_2065	*mnaA*	“	6.29	1.09 ± 0.03	**	3.21	127.71 ± 32.97	*	129.13 ± 29.51		35.67 ± 8.12		0.77 ± 0.23	*
SAUSA300_2439	*galU*	“	5.82	1.03 ± 0.07		3.2	93.7 ± 20.45		76.75 ± 21.64		24.75 ± 1.53	*	1.37 ± 0.40	****
SAUSA300_0945	*menF*	Terp/poly	5.02	1.06 ± 0.02	****	3.11	106.58 ± 25.36		101.17 ± 35.51		41.07 ± 11.03		0.62 ± 0.17	
SAUSA300_0946	*menD*	“	4.83	1.04 ± 0.04	****	3.18	93.17 ± 26.54		87.62 ± 30.22		37.75 ± 15.93		0.7 ± 0.23	
SAUSA300_1470	*ispA*	“	10.96	1.07 ± 0.01		2.93	97.5 ± 12.65		218.92 ± 39.65	****	48.34 ± 7.35	**	1.07 ± 0.38	****
SAUSA300_0504	*pdxS*	Co-f and vitamin	5.93	0.6 ± 0.09	****	4.92	30.11 ± 8.36	****	41.59 ± 8.46	****	NA		NA	**
SAUSA300_0965	*folD*	“	8.63	0.74 ± 0.05	****	5.95	93.42 ± 18.06		107.48 ± 40.20		24.18 ± 9.42		0.2 ± 0.08	**
SAUSA300_1090		Transcription	6.05	1.02 ± 0.04		2.75	88.25 ± 17.62		87.97 ± 28.27		29.92 ± 10.98		0.64 ± 0.22	
SAUSA300_1112	*stp1*	Phosphatase	4.43	1.1 ± 0.03	*	3.19	112.5 ± 25.06		126.17 ± 28.52		42.21 ± 6.64		1.29 ± 0.21	****
SAUSA300_1255	*fmtC*	Enzyme	6.7	1.12 ± 0.04		2.55	89.89 ± 28.70		193.46 ± 48.89	****	48.98 ± 12.11	**	0.8 ± 0.16	**
SAUSA300_0959	*fmtA*	“	4.8	1.11 ± 0.03	*	3.09	72.83 ± 22.09	*	175.93 ± 52.55	***	69.41 ± 17.74	****	1.21 ± 0.34	****
SAUSA300_2174	*ecfT*	ABC transport	5.44	1.05 ± 0.12		3.33	102.92 ± 11.60		102.8 ± 49.79		31.13 ± 6.64		0.63 ± 0.15	
SAUSA300_2175	*cbiO*	“	6.08	1.11 ± 0.07	*	3.08	109.83 ± 28.51		114.02 ± 14.85		46.63 ± 9.47	*	0.93 ± 0.26	*
SAUSA300_0387	*pbuX*	Transport	5.46	0.94 ± 0.01		2.14	94.44 ± 18.10		109.34 ± 21.09		16.57 ± 1.78	****	0.58 ± 0.18	
SAUSA300_0552	*bshB2*	Enzyme	4.31	0.93 ± 0.05	*	1.84	112.58 ± 27.04		87.38 ± 25.66		29.19 ± 6.91		0.52 ± 0.13	
SAUSA300_0917	*itaA*	Transport	4.97	1.06 ± 0.04	****	2.66	131.25 ± 23.58	**	139.72 ± 32.08	*	40.42 ± 4.31		0.77 ± 0.22	*
SAUSA300_0958		Enzyme	10.27	1.04 ± 0.02	****	3.06	77.89 ± 16.59		122.58 ± 33.03		23.38 ± 4.45	**	0.63 ± 0.12	

a AA, arachidonic acid; Terp/poly, terpenoid/polyketide; Co-f, co-factor.

b NA, strain did not grow.

c Significance was as follows: *, *P* < 0.05; **, *P* < 0.01; ***, *P* < 0.001; ****, *P* < 0.0001.

d OCR, oxygen consumption rate.

e ECAR, extracellular acidification rate.

Given the importance of biofilm formation during infection (22), we also examined the capacity of these mutations to form biofilms in a static microtiter assay. While we did not observe large changes in growth under these conditions (see Fig. S4 in the supplemental material), we did observe 14 mutants across multiple metabolic pathways that exhibitive defects in biofilm formation, including mutations in *pdhA*, *pyc*, *atpA*, *ilvE*, and *pdxS* (Table 2 and Fig. 3A), the majority of which grew normally under these conditions (Fig. S4). These data suggest that the ability to replicate and/or form biofilms might constitute one key dimension for how S. aureus metabolism contributes to its *in vivo* pathogenesis.

10.1128/mBio.00814-21.4 FIG S4 Growth of S. aureus mutants prior to biofilm quantification. Bacterial growth was quantified by optical density at 600 nm (OD_600_). All values (*N* = 12) provided are from three independent experiments. Bars represents means with standard deviation. ****, *P* < 0.0001; ***, *P* < 0.001; **, *P* < 0.01; *, *P* < 0.05. Download FIG S4, TIF file, 0.4 MB. Copyright © 2021 Kim et al. 2021 Kim et al. https://creativecommons.org/licenses/by/4.0/ This content is distributed under the terms of the Creative Commons Attribution 4.0 International license.

We hypothesized that the conditional essentiality of the other metabolic mutants may alternatively be due to differences in the ability to tolerate host processes such as macrophage killing (Table 2 and Fig. 3A). To further extend our macrophage survival assay data, we also assayed the ability of these 52 metabolic mutants to survive against host immune products. Arachidonic acid (AA) is released by innate immune cells during the oxidative burst and has been demonstrated to kill S. aureus through lipid peroxidation (23). We grew these 52 strains in the presence or absence of a sublethal concentration of AA to determine if defective metabolism may sensitize S. aureus to host-derived oxidative stress. We observed significantly increased sensitivity, as quantified by increased lag phase time compared to those for the untreated controls, for several metabolic mutants, including strains harboring mutations in *sucC*, *qoxC*, *cyoE*, *carA*, and *folD* (Table 2 and Fig. 3A).

The host metabolite itaconate influences biofilm formation in several other bacterial species (24) and has been shown to exhibit antimicrobial properties (25). As an independent test for the involvement of S. aureus metabolism in surviving against host stresses, we assayed biofilm formation in each of the metabolic mutants in the presence of itaconate. We observed that at physiologically relevant concentrations (26), itaconate perturbed biofilm formation (Table 2 and Fig. 3A) for many strains. Some strains were also observed to have enhanced resistance to itaconate during biofilm growth, including *fmtA*::Tn, *fmtC*::Tn, *bfmBAB*::Tn, and *ispA*::Tn mutants (Table 2). Together, these data suggested that the ability to resist host defenses might comprise a second key dimension for understanding the role of S. aureus metabolism in its pathogenesis.

### Functional phenotypic assays reveal two metabolic dimensions of S. aureus pathogenesis.

To more formally understand how these functional phenotypes may be related, we performed several statistical analyses on these data. Hierarchical clustering highlighted the relationships between genes and the assayed phenotypes (Fig. 3A). Mutants with delays to lag phase in minimal medium, sensitivity to arachidonic acid, and survival in macrophage killing assays clustered together. A significant correlation was observed between extracellular and intracellular survival, after accounting for growth defects, by strains harboring transposons in genes involved in central metabolism (Fig. 3B). Correlations were less evident in genes outside of central metabolism (Fig. 3B). Differences in intracellular survival to macrophages were not due to decreases in phagocytosis (Fig. S1B).

Consistent with our hypothesis, principal component analysis (PCA) showed that most of the variation in phenotypes for these metabolic mutants could be explained by two principal components. These components are associated with growth (principal component 1 [PC1], 31% explained variance) and stress tolerance-resistance to host defenses (PC2, 20% explained variance) (Fig. 3C). While bacterial outgrowth is not observed in the pneumonia model, it aids in preventing rapid clearance due to the host response (27). Replication is not only important from a growth perspective, but it also facilitates production of virulence factors that aid in defense against the host. We demonstrated the importance of replication *in vivo* with mice treated with the bacteriostatic antibiotic linezolid, which were cleared significantly faster than vehicle controls (see Fig. S5 in the supplemental material). To better understand these two principal components, we examined the loadings corresponding to PC1 and PC2 and found that PC1 was associated with differences in growth phenotypes (positive loadings for M9 medium lag phase delay and sensitivity to arachidonic acid during growth and negative loading for M9 medium carrying capacity), while PC2 was associated with differences in stress response phenotypes (small positive loading for biofilm formation in the presence of itaconate and negative loadings for extracellular and intracellular macrophage survival) (Fig. 3D).

10.1128/mBio.00814-21.5 FIG S5 Replication *in vivo* is important for pathogenesis. Mice were treated with linezolid prior to and during intranasal infection with S. aureus USA300. Bacterial counts were enumerated 24 h after infection. Bronchoalveolar lavage fluid, 4 data points are not on the graph, as no bacteria were detected. Each point represents a mouse. Lines display median. *N* = 6. **, *P* < 0.01; *, *P* < 0.05. Download FIG S5, TIF file, 0.1 MB. Copyright © 2021 Kim et al. 2021 Kim et al. https://creativecommons.org/licenses/by/4.0/ This content is distributed under the terms of the Creative Commons Attribution 4.0 International license.

We previously demonstrated that central carbon metabolism and cellular respiration are involved in bacterial responses to lethal stress by antibiotics (28). We therefore hypothesized that these stress tolerance phenotypes in PC2 may also be linked to cellular respiration. To test this hypothesis, we measured oxygen consumption rates (OCR) and extracellular acidification rates (ECAR) for all 52 metabolic mutants and performed correlation analyses between these respiration results and the stress tolerance phenotypes (Table 2, Fig. 3E and F; see also Fig. S6 in the supplemental material). Metabolic disruption to diverse metabolic pathways elicited significant respiratory defects in several mutants (Table 2). ECAR was significantly correlated with intracellular macrophage survival in strains that had mutations in central carbon metabolism (Fig. 3E; *R*^2^ = 0.36; *P* < 0.05), which was not evident in mutations in other metabolic genes. While OCR did not appear to have a significant correlation with survival in intracellular or extracellular macrophage killing assays (Fig. S6), OCR did appear to be significantly correlated with biofilm formation in the presence of itaconate (*R*^2^ = 0.36; *P* < 0.01) (Fig. 3F). Interestingly, metabolic mutants exhibiting increased OCR appeared to protect against itaconate stress in biofilms; these included transposon insertions in *fmtA* and *fmtC* that influence the cell wall and aid in resistance to antibiotics (29, 30). To validate these OCR measurements, we quantified ATP production in *fmtC*, *fmtA*, *fba*, and SAUSA300_1902::Tn strains and confirmed that oxygen consumption rate (OCR) values corresponded to ATP production by the cell and linked cellular respiration to biofilm formation in the presence of itaconate (Fig. 3G). Taken together, these phenotypic assays and multivariate analyses indicate that metabolism in S. aureus is important for *in vivo* pathogenesis by at least two key mechanisms, namely, (i) by supporting the ability for S. aureus cells to grow in the airway microenvironment, and (ii) by promoting tolerance to host defenses.

10.1128/mBio.00814-21.6 FIG S6 Correlation plots of macrophage killing assays with oxygen consumption rate. The WT is shown in green. Each point represents a mutant. *N* ≥ 3. Download FIG S6, TIF file, 0.2 MB. Copyright © 2021 Kim et al. 2021 Kim et al. https://creativecommons.org/licenses/by/4.0/ This content is distributed under the terms of the Creative Commons Attribution 4.0 International license.

### Pyruvate carboxylase is important across multiple infection sites.

**FIG 4:**
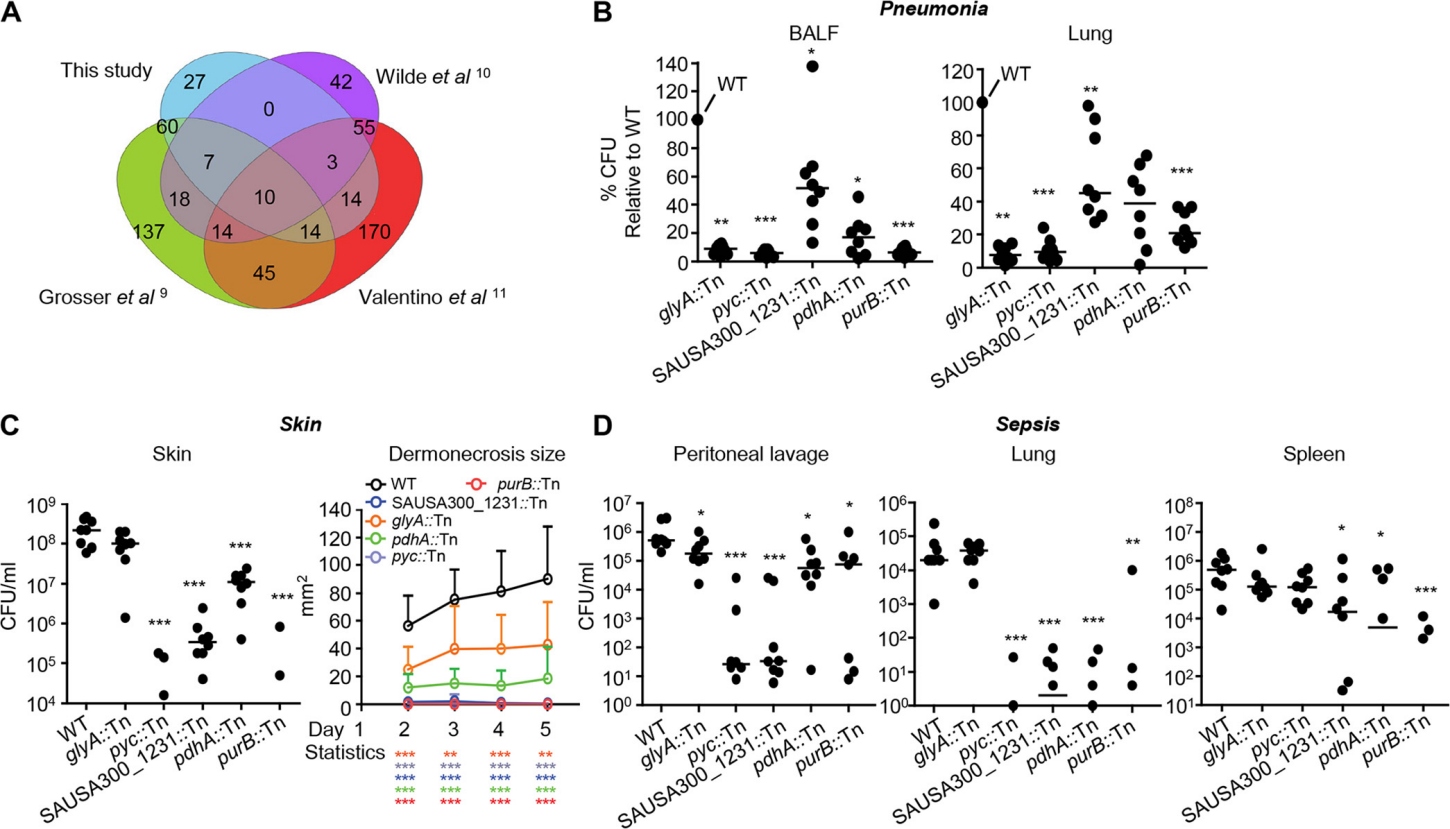
Identification and confirmation of genes from Tn-seq studies shown to be important for pathogenesis in multiple infection models. (A) Genes identified in this study as conditionally essential were compared to those in three other S. aureus Tn-seq studies conducted in models of skin infection and osteomyelitis. (B) Mice were infected intranasally with 10^7^ CFU of WT S. aureus and 5 mutants. Bacterial counts in BALF and lung tissue were quantified 24 h after infection. (C) Mice were infected subcutaneously with 10^6^ CFU of WT S. aureus and the indicated mutants. Bacterial counts were assessed from skin biopsy specimens 5 days after infection. The area of dermonecrosis was monitored daily for the duration of the experiment. (D) Mice were infected intraperitoneally with 10^7^ CFU of S. aureus. Bacterial counts in peritoneal lavage and lung and spleen tissue were enumerated 24 h after infection. *N* = 8 from at least two independent experiments. Lines display median. Data shown are the mean with standard deviation. Bacterial points below the limit of detection are not shown. ***, *P* < 0.001; **, *P* < 0.01; *, *P* < 0.05.

Concurrent with these phenotypic analyses, we bioinformatically identified genes that are potentially important across multiple infection sites through a comparison of our Tn-seq data to that from other S. aureus Tn-seq studies conducted in the skin and bone (9–11). We identified 27 mutations that are uniquely important to the airway. Of those genes with characterized functions, we identified genes associated with DNA repair (*rexA* and *rexB*), folate biosynthesis (*folA* and *thyA*), and d-alanine synthesis (*dltA* and *dltB*) as being important in the airway. We also identified 10 genes (*purB*, SAUSA300_2071, *pdhA*, SAUSA300_1231, *glyA*, *pyc*, *sucC*, SAUSA300_1255, and SAUSA300_1172) common to all studies (Fig. 4A). We confirmed that five (4 of which are metabolic) mutants (*purB*::Tn, *pdhA*::Tn, SAUSA300_1231::Tn, *glyA*::Tn, and *pyc*::Tn) were significantly attenuated in the acute pneumonia model by infecting mice with individual transposon mutants (Fig. 4B). Both *pdhA* and *pyc* products are involved in flux into the TCA cycle; *pdhA* encodes a pyruvate dehydrogenase, while *pyc* encodes a pyruvate carboxylase that converts pyruvate to oxaloacetate. The gene *purB* encodes an adenylosuccinate lyase, which functions in the conversion of IMP to AMP in purine biosynthesis, and *glyA* encodes a serine hydroxymethyltransferase, which is involved in the conversion of serine to glycine. The fifth gene identified, SAUSA300_1231, is a predicated γ-aminobutyrate permease. We also determined the importance of these genes during skin and septic infection. All mutants were significantly attenuated in the murine model of skin infection (Fig. 4C). The *pyc* mutant was the most attenuated and did not display regions of dermonecrosis. The bacterial counts of the *pyc* mutant were reduced by over 5,700-fold (*P* < 0.001), adding to its attenuated phenotype during bone infection (31). Each mutation was also significantly attenuated in a model of systemic infection (Fig. 4D). Infection severity was significantly decreased for all mutants (*P* < 0.05; Fig. 4D) compared to that of the WT in at least one location (peritoneum, lung, or spleen). Strains SAUSA300_1231::Tn, *purB*::Tn and *pdhA*::Tn had significant reductions in bacterial burdens in the peritoneum, lung, and spleen (*P* < 0.05; Fig. 4D), while the *pyc* mutant had bacterial burdens over 10,000-fold less in peritoneal lavage fluid and the lung (Fig. 4D). Thus, mutations in SAUSA300_1231, *pyc*, *purB*, *pdhA*, and *glyA* represent genes important for pathogenesis across all models of infection and highlight the importance of purine and glycine metabolism, γ-aminobutyrate transport, and flux into the TCA cycle during infection.

### Pyruvate carboxylase contributes to cellular respiration and growth.

Given that several experiments found that the absence of *pyc* had a significant impact on pathogenesis, we chose to study pyruvate carboxylase in more detail for deep validation of our data. We genetically complemented the mutation through stable chromosome integration. Pyruvate carboxylase is the major anaplerotic enzyme that carboxylates pyruvate to enter the TCA cycle. Its activity is necessitated by the requirement to replenish TCA cycle intermediates that are removed for other functions, such as gluconeogenesis and fatty acid synthesis.

**FIG 5:**
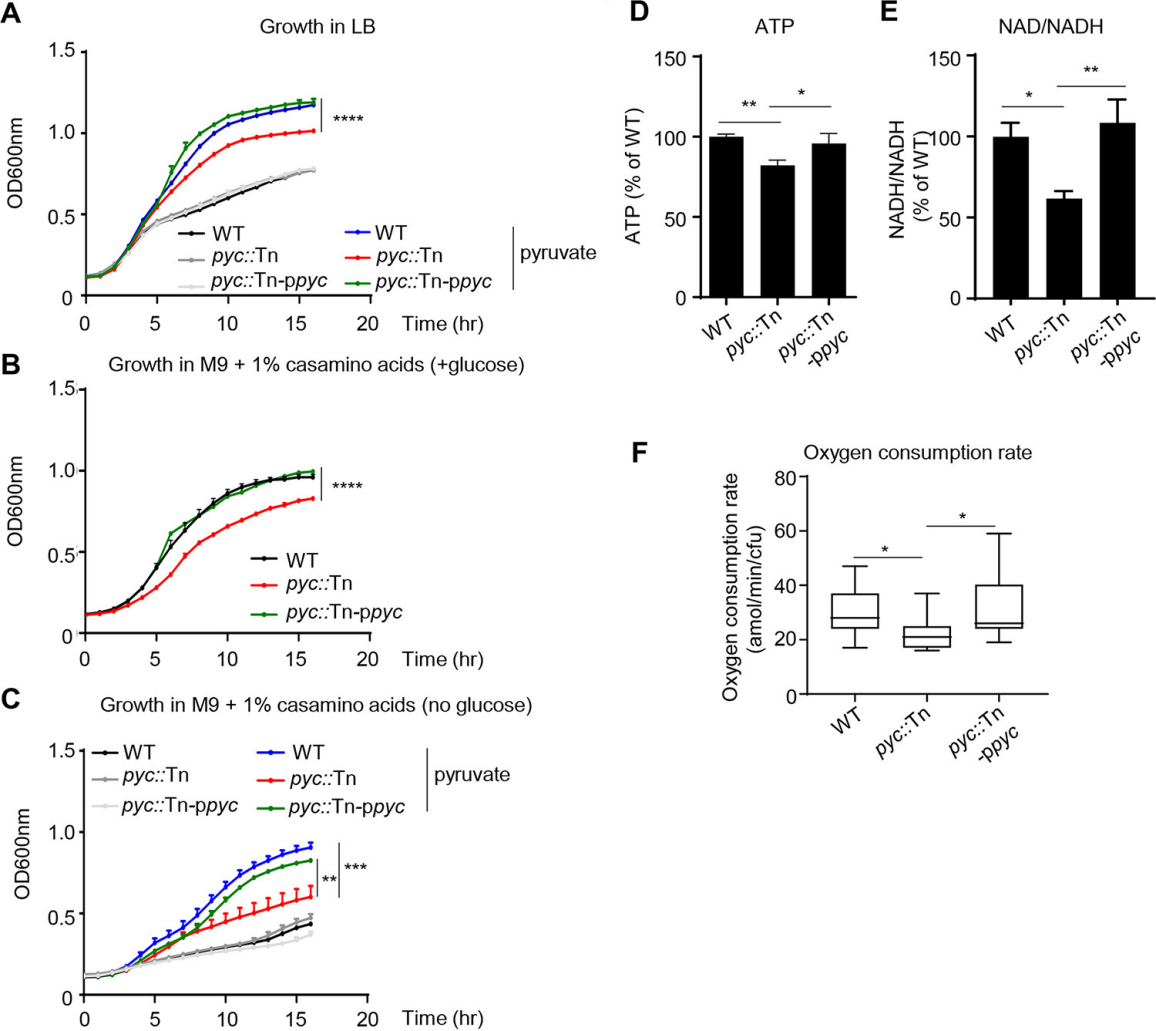
Pyruvate carboxylase contributes to growth and respiration. Bacterial growth in (A) LB broth (with or without 2% pyruvate), (B) M9 medium with 1% Casamino Acids and glucose, or (C) M9 medium (without glucose) with 1% Casamino Acids (with or without 2% pyruvate) was detected by optical density at 600 nm (OD_600_) for 16 h. *n* = 6. (D) ATP and (E) NAD/NADH concentration of cultures grown to the exponential phase. *n* = 7. (F) Oxygen consumption rates (OCR) measured by Seahorse analysis. *n* = 17. All data shown are from at least two independent experiments. Bars represent means with standard deviation. ***, *P* < 0.001; **, *P* < 0.01; *, *P* < 0.05.

**FIG 6:**
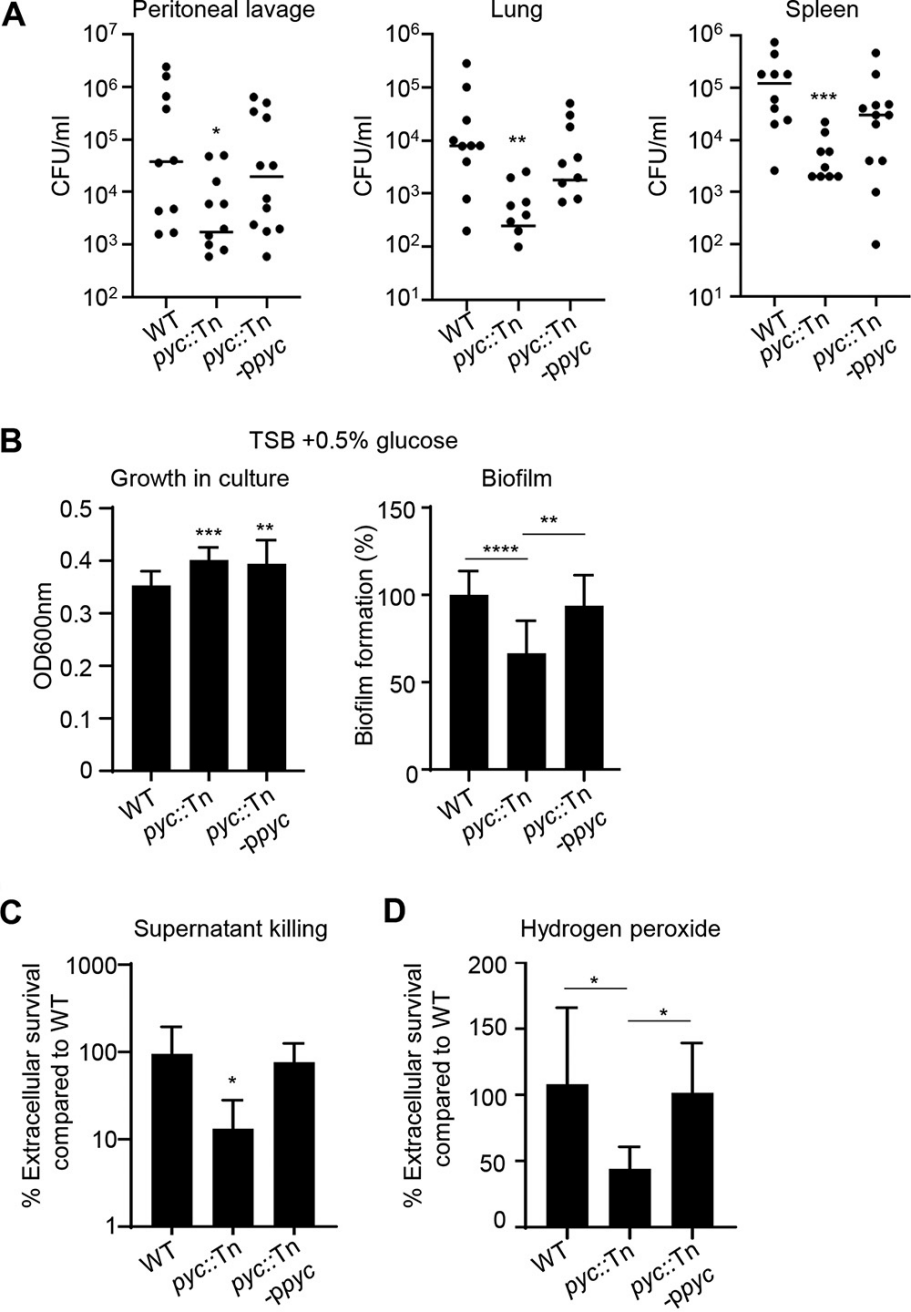
Pyruvate carboxylase contributes to pathogenesis and protection from host cell killing. (A) Mice were infected intraperitoneally with 10^7^ CFU of WT (*n* = 10), *pyc* mutant (*n* = 12), or *pyc*-complemented (*n* = 12) strains. Bacterial counts in peritoneal lavage fluid and lung and spleen tissue were enumerated 24 h after infection. (B) Bacterial growth and biofilm formation (*n* = 18). (C) Bone marrow-derived macrophage (BMDM) culture supernatant was incubated with S. aureus for 4 h and surviving bacteria were determined. *n* = 7. (D) Killing after exposure to hydrogen peroxide (0.5%) for 2 h. *n* = 7. All data shown are from at least two independent experiments. Bars represent means with standard deviation. ****, *P* < 0.0001; ***, *P* < 0.001; **, *P* < 0.01; *, *P* < 0.05.

From our PCA, the first metabolic dimension associated with virulence was growth (Fig. 3D and E). We therefore examined the pyruvate carboxylase mutant for defects in bacterial growth and energy production. Under nutrient-rich (LB medium) conditions, there was no impact on bacterial growth of the *pyc*::Tn strain (Fig. 5A). However, growth differences could be observed in the presence of exogenous supplementation with pyruvate; the addition of pyruvate increased growth of WT S. aureus by 45% (*P* < 0.0001; Fig. 5A), while in the absence of *pyc*, growth only increased by 24% (*P* < 0.0001; Fig. 5A). Growth of the *pyc*::Tn strain was reduced in defined medium (M9 plus Casamino Acids) using glucose as a carbon source (Fig. 5B). This growth defect was further exacerbated (32% reduction; *P* < 0.001; Fig. 5C) compared to the WT strain when glucose was replaced by pyruvate. Returning *pyc* restored growth of the *pyc*::Tn strain to normal. Indicative of its observed growth defect and role in the TCA cycle, we observed that *pyc*::Tn had significantly reduced ATP activity (18% decrease; *P* < 0.001; Fig. 5D) and a 39% decrease in NAD/NADH production (*P* < 0.05; Fig. 5E). Furthermore, the oxygen consumption rate was decreased in the absence of *pyc* (Fig. 6F and Table 2).

### Contribution of pyruvate carboxylase to S. aureus pathogenesis.

We confirmed the role of pyruvate carboxylase during infection by using our genetically complemented strain and the *pyc* mutant with our murine sepsis model, which showed the most profound defect in infection compared to pneumonia and skin models (Fig. 2D and E and Fig. 4). Returning *pyc* to the *pyc*::Tn mutant restored virulence in this model (Fig. 5A). No differences in immune cell populations were observed (see Fig. S7 in the supplemental material. As inactivation of *pyc* led to growth and metabolic defects, we next examined its resistance to host processes and biofilm formation, since these were revealed by PCA to be key phenotypes (Fig. 3E). The observation that a mutation in *pyc* led to attenuation in the absence of differences in immune cell numbers suggested that the *pyc* mutant strain had a growth defect or an impaired capacity to defend against the host response. We predicted that an absence of anaplerosis would impair biofilm formation by S. aureus. The absence of *pyc* led to a 33% decrease in biofilm formation (*P* < 0.0001), which was restored by genetic complementation (Fig. 6B). Confirming our RAW cell data (Table 1), mutation of *pyc* led to a significant defect in survival when it was exposed to bone marrow-derived macrophages (BMDMs) (see Fig. S8 in the supplemental material), with comparable survival to that of the WT in the intracellular assay (Fig. S8). Macrophage survival could be due to altered adherence to macrophages or sensitivity to a secreted product. We observed that the *pyc*::Tn mutant was killed 87% (*P* < 0.05) more effectively than the WT strain when exposed to spent macrophage supernatant (Fig. 6C); this indicates the strain is more sensitive to the decreased nutrients in the spent supernatant or secreted antimicrobial products from the macrophages. The latter point was evident by demonstrating the enhanced susceptibility of *pyc* to hydrogen peroxide (Fig. 6D). These data validate our findings linking *in vivo* attenuation to both growth and resistance to host killing mechanisms for genes involved with S. aureus metabolism.

10.1128/mBio.00814-21.7 FIG S7 Flow cytometry analysis of cells in peritoneal lavage fluid from sepsis infection. WT C57BL/6J mice were infected with WT S. aureus USA300, the *pyc* mutant, and the *pyc*-complemented strain. DC, dendritic cell; PMN, polymorphonuclear leukocytes/neutrophils; pDC, plasmacytoid dendritic cell; PM, peritoneal macrophage; NK, natural killer cell; IM, interstitial macrophage. Graphs display mean with standard deviation. Download FIG S7, TIF file, 0.3 MB. Copyright © 2021 Kim et al. 2021 Kim et al. https://creativecommons.org/licenses/by/4.0/ This content is distributed under the terms of the Creative Commons Attribution 4.0 International license.

10.1128/mBio.00814-21.8 FIG S8 Deletion of *pyc* reduces survival against macrophages. Extracellular survival and intracellular gentamicin protection assay with bone-marrow derived macrophages (MOI = 1). *n* = 9. Graphs show means with standard deviation. *, *P* < 0.05. Download FIG S8, TIF file, 0.1 MB. Copyright © 2021 Kim et al. 2021 Kim et al. https://creativecommons.org/licenses/by/4.0/ This content is distributed under the terms of the Creative Commons Attribution 4.0 International license.

## DISCUSSION

In this study, we identified 136 genes associated with pathogenesis of S. aureus in the lung using Tn-seq. Through this large-scale mutant screening approach, we were able to identify phenotypes for many of these genes through both *in vitro* and *in vivo* assays. The majority of the genes identified have not previously been described for their importance in airway colonization, and this list included many uncharacterized hypothetical genes. By comparing this study to others, including three different infection models, we were able to identify several genes that contributed to pathogenesis across multiple infection models. The importance of metabolic genes *in vivo* was apparent, with central metabolism connected with growth and survival against host cell killing.

This study represents a major contribution to understanding the pathogenesis of S. aureus in the airway. While much has been done to define the various factors of S. aureus important for causing disease, few have been tested and validated as playing a role in the pathogenesis of S. aureus pneumonia. S. aureus produces many toxins, including alpha-toxin and beta-toxin, which play primary roles during infection (5). We now have over 100 genes that we can potentially define as playing a role in pulmonary infection. We individually validated 23 of these genes as having a role in infection. We also identified many genes that had no previously known function in S. aureus or beyond the genus. Of the conditionally essential 136 genes, 11 hypothetical genes were identified, and 6 genes were confirmed as being attenuated *in vivo*. While our *in vivo* and Tn-seq studies ascribed some function to these genes, further studies will be required to address the functions of the open reading frames (ORFs) and to better understand their role during infection.

We utilized several bacterial and host-based assays to identify mechanisms behind the genes that were identified from the Tn-seq screen. Our assays were based upon the demonstrated requirement for these attributes in lung infection, such as hemolytic activity and survival with macrophages (20, 22, 32, 33), but were also applicable to various sites of infection. Not only did we identify mutations that had distinct properties, such as reduced hemolysis, defective biofilm formation, or decreased respiration, it allowed us to make some much larger observations. Through our multivariate analysis, we were able to draw the connection between growth in minimal medium and survival against macrophage killing. We took this a step further and learned from PCA that most of the data could be explained by two principal components that appear to be characterized by (i) bacterial growth and biofilm formation and (ii) tolerance to host stresses. These two elements were strongly linked for mutants in central carbon metabolism, and their growth defects presumably influenced their survival to stresses. However, outcomes such as growth and macrophage survival were uncoupled in mutants outside central carbon metabolism, including properties that might enhance their resistance to host stresses, such as those we observed with the *pyc*::Tn mutant and itaconate. The uncoupling of these variables in other metabolic genes can be seen with the *menD*::Tn mutant with decreased growth but enhanced macrophage survival, the *bfmBAB*::Tn mutant with enhanced biofilm but attenuated growth in minimal medium or enhanced biofilm formation with *ugtP*::Tn without changes in growth, or the *ispA*::Tn mutant, which grew normally but had increased resistance to itaconate but also enhanced sensitivity to arachidonic acid. While these two general categories accounted for most of the variance in data (∼60%), not all factors were accounted for in this study. Future directions include identifying the mechanisms behind the attenuation in virulence of these strains. One interesting correlation was that oxygen consumption rate appeared to be correlated with tolerance to the host metabolite itaconate during biofilm formation. Through rigorous screening and analysis, we are able to understand why many mutations led to attenuation *in vivo*; however, the mechanism of pathogenesis is still to be determined for many genes.

Large-scale studies of S. aureus infection using Tn-seq have been conducted in skin abscess and bone models of infection (9–11, 13). Studies have also been conducted that examined what happens to S. aureus essential genes in the context of polymicrobial infection (34). Under coinfecting conditions, many genes are no longer essential, while others become essential. We also observed in our Tn-seq screen an absence of some previously characterized virulence factors in the airway, such as alpha-toxin and protein A, which may have not shown up due to cross-complementation among the transposon mutant pool. Comparison of the other monoinfection Tn-seq studies facilitated the identification of 10 genes across all studies that were important in all models of infection. From these 10 genes, we confirmed five to be important in multiple infection models, namely SAUSA300_1231, *pyc*, *purB*, *pdhA*, and *glyA*. Among these, *purB* is known to be involved in purine biosynthesis (35), and purine metabolism has been shown to be important for virulence (13, 36, 37). Both *purB* and *pdhA* mutants were attenuated in our *in vitro* killing assays. Related to *pdhA* and *pyc* mutants, it has been shown that pyruvate has an important role in the regulation of virulence (38), while a previous role in virulence has not been attributed to *glyA* or SAUSA300_1231. Pyruvate carboxylase was recently shown to be important during bone infection, contributing to aspartate biosynthesis (31). While absence of *pyc* led to a significant defect against macrophages and their products, it also allowed the mutant to persist against itaconate, indicating a potential balance between growth deficiency and features making it more resistance to host stress. It would be interesting to examine if there is an association between ATP levels and antibiotic resistance, as has been observed with persister cells (40). So, while in the absence of *pyc*, S. aureus is less pathogenic, *in vivo* it may possess qualities to persist in the context of a chronic infection.

This study is one of only a few that have investigated the bacterial genetic requirements for infection in the airway. A screen of the genetic requirements for Acinetobacter baumannii (41) in the airway identified a large metabolic requirement analogous to that observed in this study, including nucleotide transport and metabolism and purine metabolism. The authors also observed a requirement for the ClpXP protease (42, 43), which we also observed (see Table S2b in the supplemental material). Genes required for transmission of Streptococcus pneumoniae (44) have been examined in ferrets and mice. In ferrets, genes associated with metabolism, competence, and metal uptake were identified, while in mice the study focused on surface proteins, such as adhesins, and proteins that interact with host glycoconjugates (45). As we were not looking at upper respiratory tract colonization and transmission, we did not see adhesins in our screen. We did not observe a requirement for some well-characterized virulence factors, and this was also observed in a Tn-seq study of respiratory melioidosis. In that study, a large number (>1,400) were identified, including type III secretion and capsule, but many previously characterized, such as lipopolysaccharide (LPS), were not identified in the screen. These studies highlight the diversity in genetic requirements with different pathogens (46).

Through our unbiased Tn-seq screen, we identified 136 genes important for S. aureus survival in the airway. The vast majority of these genes had not been previously associated with survival in the lung. We highlight an important role for central metabolism in growth and resistance to host-mediated immune cell killing and identified two major components that account for virulence, namely the ability to grow under limiting conditions and form biofilms, and tolerance to different host stresses. These processes were tightly associated with central metabolism, while they could be uncoupled for other metabolic pathways that were attenuated through additional mechanisms. As deep validation for our observations, we also demonstrated an important role for pyruvate carboxylase in pathogenesis and the balance between host susceptibility and potential to persist against host antimicrobial compounds. Conventional understanding of pathogenesis in the context of host-pathogen interactions is understood in term of factors that can neutralize the host immune response. We demonstrate that innate features of microbial metabolism are key requirements for *in vivo* infection, independent from host immunomodulation, and provide a basis for identification of several new targets for antimicrobial therapy and vaccine design.

## MATERIALS AND METHODS

### Bacterial strains and cell lines.

The S. aureus USA300 (LAC) transposon library used for transposon sequencing was a kind gift from Anthony Richardson (9), while individual S. aureus transposon mutants (7, 8) used for screening were on the JE2 USA300 background from the Nebraska Transposon Mutant Library (NTML) (8). Mutations were confirmed using PCR and quantitative reverse transcription-PCR (qRT-PCR) (see Table S1 in the supplemental material). S. aureus strains used in this study were grown in Luria-Bertani (LB) broth at 37°C with 300 rpm shaking until they reached an optical density at 600 nm (OD_600_) of 1.0. Murine macrophage cells (RAW 264.7, provided by George Yap) were cultured in Dulbecco’s minimal essential medium (DMEM) supplemented with 10% fetal bovine serum and 1% antibiotics (penicillin and streptomycin). Bone marrow-derived macrophages (BMDMs) were generated as described previously (27).

10.1128/mBio.00814-21.9 TABLE S1 Primers for PCR and quantitative reverse transcription-PCR (qRT-PCR) for confirmation of mutants in this study. Download Table S1, DOCX file, 0.01 MB. Copyright © 2021 Kim et al. 2021 Kim et al. https://creativecommons.org/licenses/by/4.0/ This content is distributed under the terms of the Creative Commons Attribution 4.0 International license.

Complemented strains were constructed using the vector pLL39 before being transformed into RN4220 containing pLL2787 and integrated onto the chromosome at the L54a *attB* site as previously described (47, 48). Episomal integration was verified using the Scv8 and Scv9 primers (47). The pLL39 episomes, including an empty vector, were individually transduced from RN4220 into the appropriate strain using bacteriophage 80α (49, 50). All bacterial strains were constructed in the JE2 genetic background and were PCR verified to ensure an insertion in the L54a *attB* site and a transposon insertion in the gene of interest.

### Tn-seq preparation and analysis.

A vial of S. aureus USA300 Tn library (9) was grown overnight in LB broth prior to a 1:100 dilution and grown to the exponential phase. Mice were intranasally infected with 2 × 10^7^ CFU of the library, and 24 h later, lungs were removed and homogenized through 40-μm filters. Lung homogenates were then outgrown for 5 h in LB at 37°C. The library was prepared for sequencing following the protocol of Grosser et al. (9) and using a Covaris sonicator that generated 300- to 700-bp-sized DNA fragments. In brief, sheared DNA was purified prior to the addition of 3′-poly(dC) tails. Nested PCR was then used to first amplify transposon junctions and to incorporate Illumina index primers to facilitate multiplexing. PCR products were purified using AMPure XP beads (Beckman) prior to sequencing using an Illumina HiSeq 2500 instrument (NYU Genome Technology Center).

Cutadapt (51) was used to remove 3′ nonspecific nucleotide reads, and sequences were filtered to remove any aberrant short (<20-nt) or long (>52-bp) reads. Processed reads were end-to-end aligned to the S. aureus USA300 reference genome using Bowtie 2 (52). The resulting alignment files were used to determine essential and conditionally essential genes with two different bioinformatics packages, ARTIST (53) and ESSENTIALS (54). The cutoff for gene essentiality in ESSENTIALS was determined using the local minimum (−5.26) of the log_2_ fold change value of detected-versus-expected transposon insertions per gene, plotted against kernel density as described previously (54). Conditionally essential genes were similarly determined using a local minimum (−3.1) of the log_2_ fold change value of detected-versus-expected transposon insertions in the mouse samples compared to the input control transposon library. Fitness calls with ARTIST were made using a *P* value cutoff of 0.05. Each of the five biological replicates from the mouse experiment were analyzed separately, and genes that ARTIST determined as conditionally essential in at least three samples were included in the ARTIST set of conditionally essential candidates. Final essentiality and conditional essentiality calls were determined based on output from the two software packages.

### Killing assays.

RAW (incubated with DMEM with 10% serum) cells or BMDM (incubated with RPMI 1640 with 10% serum) with antibiotics removed were incubated with S. aureus at a multiplicity of infection (MOI) of 10 for 2 h at 37°C before medium was removed and cells were serially diluted onto LB agar plates to determine extracellular bacterial killing. S. aureus incubated with DMEM with 10% serum in the absence of macrophages served as a control and was used to normalize the extracellular and intracellular survival data. Data were analyzed by comparing mutant growth in the presence or absence of macrophages prior to comparison to the WT control. We did not observe any differences in bacterial growth in the presence or absence of serum. To detect intracellular bacteria, cells were incubated with S. aureus at an MOI of 1 for 2 h, then washed and treated with 500 μg/ml of gentamicin for 2 h. Cells were then washed with phosphate-buffered saline (PBS), detached with 100 μl of TrypLE Express (Invitrogen), and serially diluted onto LB agar plates. Sensitivity to hydrogen peroxide was conducted on exponential-phase bacteria grown in LB (OD_600_ = 1.0), diluted 1:100 from overnight cultures. S. aureus was incubated with 0.5% hydrogen peroxide in PBS for 2 h, then serially diluted onto LB agar plates to determine surviving bacteria. The macrophage supernatant assay was undertaken by incubating S. aureus (10^7^) for 4 h with clarified BMDM supernatant at day 7 of differentiation. Data were analyzed by comparing mutants to the WT strain for supernatant killing. Bacteria were enumerated through serial dilution and culturing on LB agar plates.

### Growth curves.

Growth assays were conducted in LB broth or M9 minimal medium with 1 mM magnesium chloride, 50 nM calcium chloride, 1% Casamino Acids, and 0.2% glucose. Growth was assessed with the addition of pyruvate (2%) or arachidonic acid (20 μM). Overnight cultures were diluted 1:100, and optical density of growth was measured for 16 h with 15-min intervals at 37°C using a SpectraMax i3x (Molecular Devices) or an Infinite M Plex (Tecan) plate reader. The time taken to exit from the lag phase was calculated for each strain by fitting a 4-parameter logistic curve to each set of OD_600_ measurements in Prism 8 (GraphPad Software, San Diego, CA) and calculating the time taken to reach 10% of log_10_ (maximum OD_600nm_) for arachidonic acid. The time taken to reach an OD_600_ of 0.2 was used for M9 medium exit from the lag phase, to account for differences between strains in maximum OD_600nm_ observed.

### Principal-component analysis.

Principal-component analysis (PCA) was performed in MATLAB (Mathworks, Natick, MA) using custom scripts. Data from phenotypic assays were first normalized by measurements in the WT control strain. A Box-Cox transformation was then performed to transform these data into normal distributions for each assay (55). A Z-score transformation was applied to the resulting data to standardize measurements between assays and PCA scores, and loadings were computed using the *pca* function in MATLAB. Heatmaps were generated using ClustVis (56).

### Oxygen consumption rate measurements.

Bacterial respiratory activity was quantified using the Seahorse XFe96 extracellular flux analyzer (Agilent Technologies, Santa Clara, CA) as previously described (28, 57). Overnight cultures of S. aureus cells in LB were diluted 1:100 in M9 minimal medium with 1% Casamino Acids and grown to the mid-exponential phase at 37°C. Cultures were diluted to an OD_600_ of 0.05, and 200 μl of diluted cells was dispensed into each well of XFe cell culture microplates precoated with 100 ng/ml of poly-d-lysine in technical triplicate. CFU were plated for reach sample. Microplates were centrifuged for 10 min at 1,600 relative centrifugal force (RCF) to adhere cells to XFe microplates. Measurements were taken at 5-min intervals with a cycle of 2.5 min of measurement and 2.5 min of mixing, with measurements averaged over the first three cycles. OCR and ECAR values displayed are normalized by CFU and averages across *n* ≥ 3 biological replicates for each strain.

### ATP and NAD/NADH quantification.

ATP levels were measured using the BacTiter-Glo microbial cell viability assay (Promega) and NAD/NADH levels were measured using NAD/NADH-Glo assay (Promega) according to manufacturer’s instructions. Diluted overnight cultures were grown to an OD_600_ of 1.0 at and resuspended with PBS.

### Cell viability and phagocytosis.

After assays and cellular detachment, trypan blue was added to cells following enumeration on a Countess II cell counter (Life Technologies). Phagocytosis assays were conducted at an MOI of 1 with Alexa Fluor 647 *N*-hydroxysuccinimide (NHS) ester (Invitrogen)-labeled strains for 5 min and 30 min at 37°C. Cells were then washed 3 times and detached with TrypLE Express. Cells were mixed with PBS and analyzed using an Accuri C6 flow cytometer (BD Biosciences).

### Hemolysis assay.

Clarified bacterial supernatants from overnight cultures were filter sterilized and incubated with 5% (wt/vol) red blood cells (sheep) in Hanks’ balanced salt solution (HBSS) for 30 min at 37°C. After 30 min of incubation, red blood cells were removed by centrifugation at 2,000 rpm in V-bottomed plates. Supernatants were then quantified at 415 nm. PBS and 10% Triton X-100 were used as negative and positive controls, respectively.

### Western blot analysis.

Bacterial cell pellets from exponential-phase cultures were resuspended with 50 μl of Laemmli sample buffer (Bio-Rad), boiled, and used for Western blotting. The samples were probed with protein A (Sigma) antibody diluted 1:10,000 in Tris-buffered saline with Tween 20 (TBST) with 5% skim milk and then probed with anti-mouse-horseradish peroxidase (HRP) (Santa Cruz Biotechnology) diluted 1:10,000 in TBST with 5% skim milk.

### PCR analysis.

S. aureus mutants were grown in LB broth until an optical density at 600 nm of 1.0. DNA was extracted using the DNeasy extraction kit (Qiagen). PCR was performed using the EmeraldAmp PCR master mix (TaKaRa). For qRT-PCR, total RNA was extracted using the E.Z.N.A. RNA kit (Omega Bio-tek), and cDNA was synthesized using random hexamers and reverse transcriptase (Applied Biosystems). Quantitative reverse transcription-PCR was performed using SYBR green (Applied Biosystems) on a QuantStudio 6 real-time PCR machine.

### Biofilm assay.

Bacterial cultures were diluted 1:100 from exponential-phase culture (OD_600_ = 1.0) in Trypticase soy broth (TSB) medium with 0.5% glucose in 96-well tissue culture plates. Fumaric and itaconic acid stocks were brought to neutral pH with NaOH. Bacterial cultures were grown for 24 h at 37°C, and growth was measured (OD_600_). Plates were washed twice with water, dried, and stained with 1% crystal violet. After 30 min, plates were washed three times with water and dried before dissolving crystal violet in 100% ethanol and measuring OD at 590 nm.

### Mouse studies.

In the pneumonia model, 6-week-old C57BL/6J mice were intranasally infected with 2 × 10^7^ to 4 × 10^7^ CFU of S. aureus in 50 μl of PBS with anesthesia (ketamine and xylazine). Twenty-four hours later, bronchoalveolar lavage fluid (BALF) was collected by washing the airway 3 times with 1 ml of PBS, and lung tissue was homogenized to enumerate bacterial counts. The skin infection model was conducted as previously described (42) with 2 × 10^6^ to 4 × 10^6^ CFU in 100 μl PBS injected subcutaneously to mice. In the sepsis model, mice were intraperitoneally infected with 2 × 10^7^ to –4 × 10^7^ CFU of S. aureus in 100 μl PBS. Mice were infected for 24 h before peritoneal lavage was collected by washing with 3 ml of PBS; lungs and spleens were homogenized to enumerate bacterial counts. Treatment with linezolid (100 mg/kg; 5% dimethyl sulfoxide [DMSO] in PBS) was every 12 h, starting 16 h before infection. Bacterial counts were quantified by using CHROMagar S. aureus plates (BD Biosciences).

### Flow cytometry.

Peritoneal lavage cells were stained with fluorescently conjugated antibodies to MARCO-fluorescein isothiocyanate (FITC) (MCA1849F; Bio-Rad), CD11c^−^ BV605 (N418), CD86^−^ BV421 (GL-1), CD103^−^ BV510 (M290; BD Biosciences), CD11b^−^ phycoerythrin (PE)-Cy7 (M1/70), Ly6G-PerCP Cy5.5 (1A8), F4/80-APC (BM8), CD45^−^ AF700 (30-F11), MHCII-APC-Cy7 (M5/114.15.2), CD200R^−^ PE (123908), Ly6C-PE-Texas Red (AL-2; BD Biosciences), and NK1.1-BV650 (PK136) and collected on a Fortessa flow cytometer (BD Biosciences). Viability was assessed using 4′,6-diamidino-2-phenylindole (DAPI). Antibodies were purchased from BioLegend unless otherwise stated. Cells were gated based on live cells and small peritoneal macrophages (CD45^+^ F4/80^+^ CD11c^−^ CD11b^+^ MHCII^+^), large peritoneal macrophages (CD45^+^ F4/80^+^ CD11c^−^ CD11b^+^ MHCII^−^), interstitial macrophages (CD45^+^ Ly6C^−^ F4/80^−^ CD11b^+^ CD11c^+^ MHCII^+^), Ly6C-negative monocytes (CD45^+^ Ly6C^−^ F4/80^−^ CD11b^+^ CD11c^+^ MHCII^−^), CD103^+^ dendritic cells (DC) (CD45^+^ Ly6C^−^, F4/80^−^, CD11b^−^, MHCII^+^, CD103^+^ CD11c^+^), C11b^+^ DC (CD45^+^ Ly6C^+^ F4/80^−^ CD11b^+^ MHCII^+^ CD11c^+^), neutrophils (CD45^+^ Ly6C^+^ F4/80^−^ CD11b^+^ MHCII^−^ Ly6G^+^), Ly6C-positive monocytes *CD45^+^ Ly6C^+^ F4/80^−^ Ly6G^−^ CD11b^+^ MHCII^−^), plasmacytoid DC (CD45^+^ Ly6C^+^ F4/80^−^ CD11b^−^ CD11c^+^ MHCII^+^), and natural killer cells (CD45^+^ Ly6G^−^ F4/80^−^ NK1.1^+^).

### Ethics statement.

Animal work was performed according to the Guidelines for the Care and Use of Laboratory Animals of the National Institutes of Health, the Animal Welfare Act, and U.S. federal law. Protocols were approved by the Animal Care and Use Committee of Rutgers New Jersey Medical School (approval no. 201800040 and 201800192).

### Statistics.

Statistics were performed with Prism software (GraphPad Software). The nonparametric Mann-Whitney test was used for animal data. Multiple comparisons were conducted using one-way analysis of variance (ANOVA) with Bonferroni’s multiple-comparison test. Graphs display means with standard deviations, and all experiments were performed at least twice with multiple independent biological replicates. A *P* value of <0.05 was considered statistically significant.

### Data availability.

Tn-seq reads were submitted to the SRA database (BioProject number PRJNA577707).
