# Supplementary figures and images for: Growth and Stress Tolerance Comprise Independent Metabolic Strategies Critical for Staphylococcus aureus Infection
Source: mBio. 2021 Jun 8;12(3):e00814-21. doi: 10.1128/mBio.00814-21 (PMC8262855; doi:10.1128/mBio.00814-21)

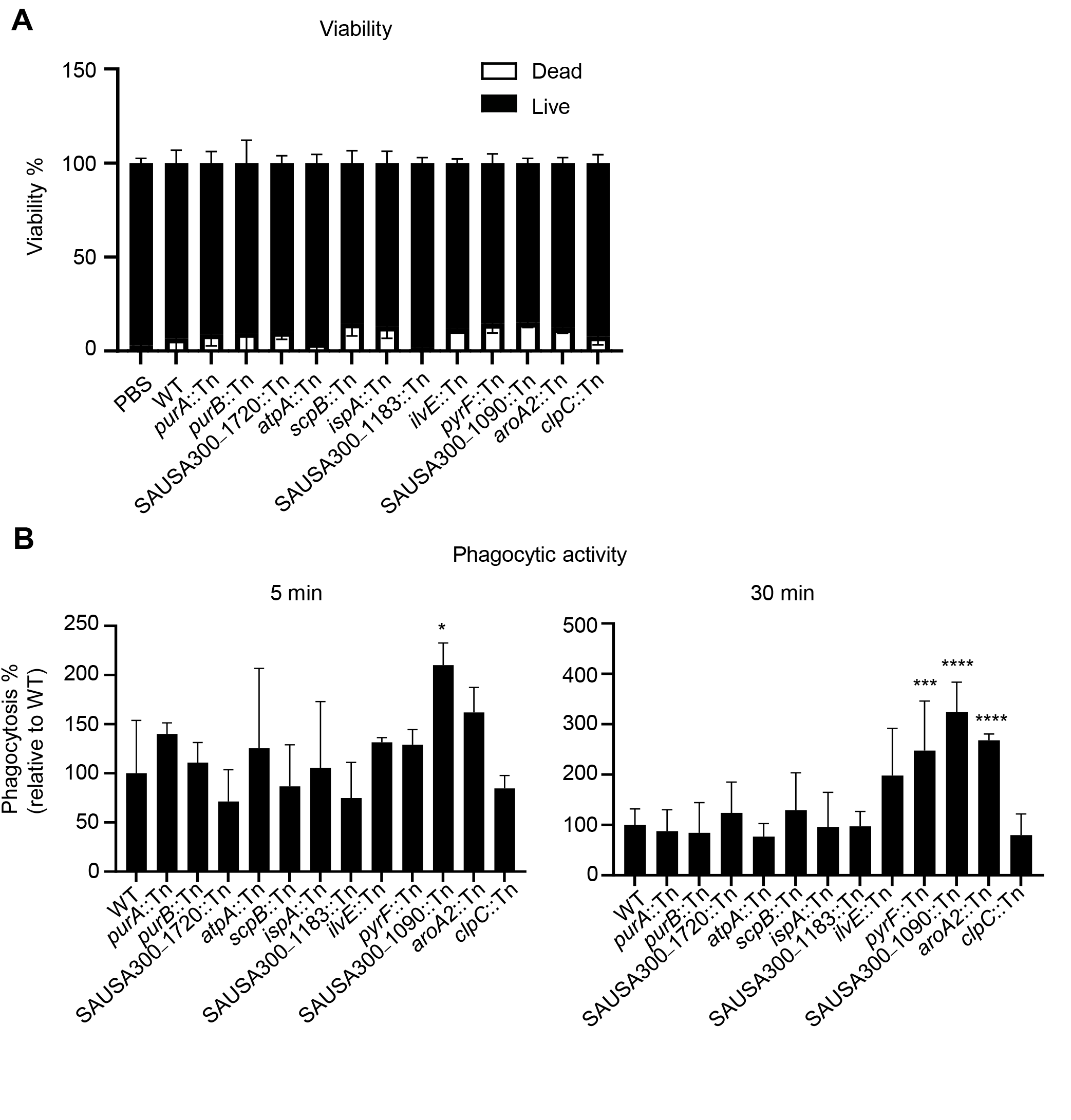

Supplement: FIG S1 [file mbio.00814-21-sf001.tif]

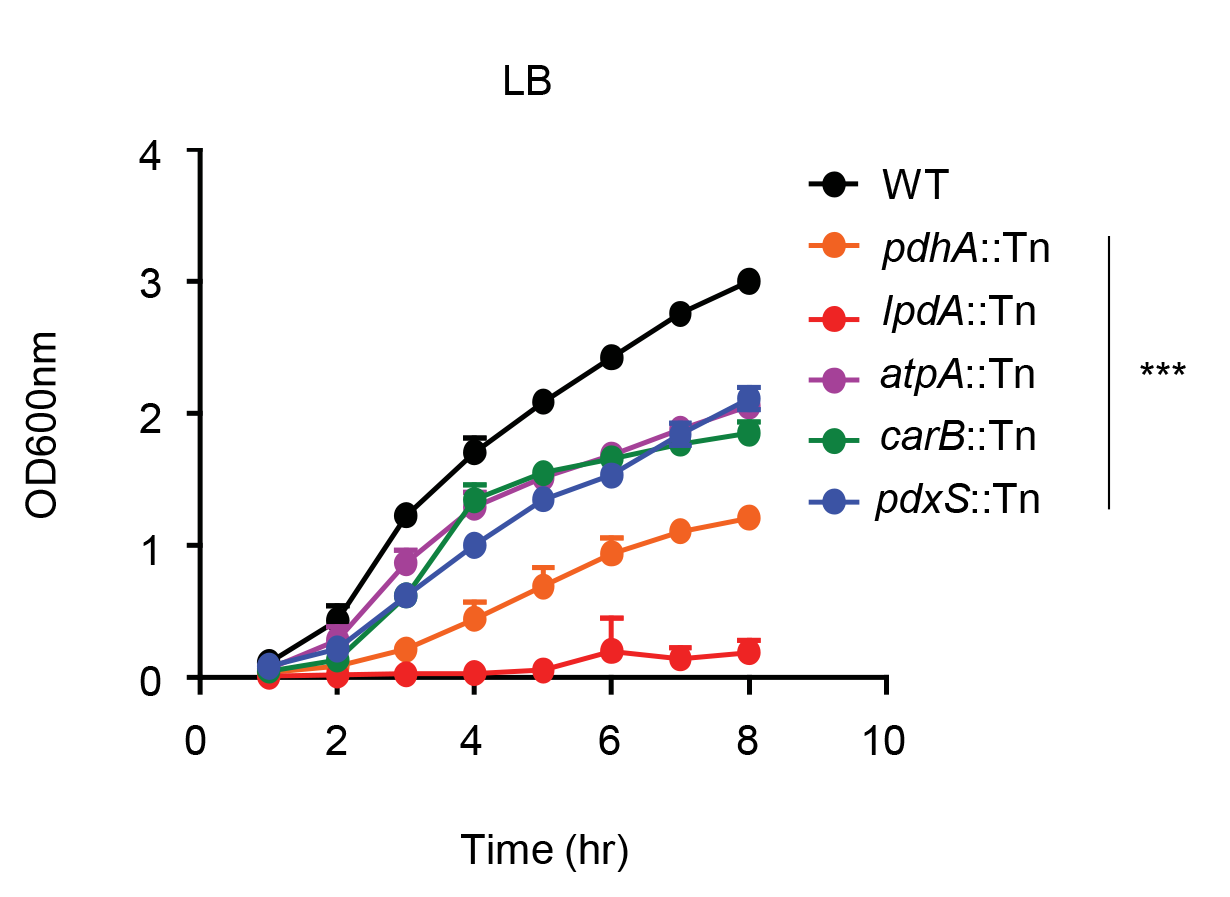

Supplement: FIG S2 [file mbio.00814-21-sf002.tif]

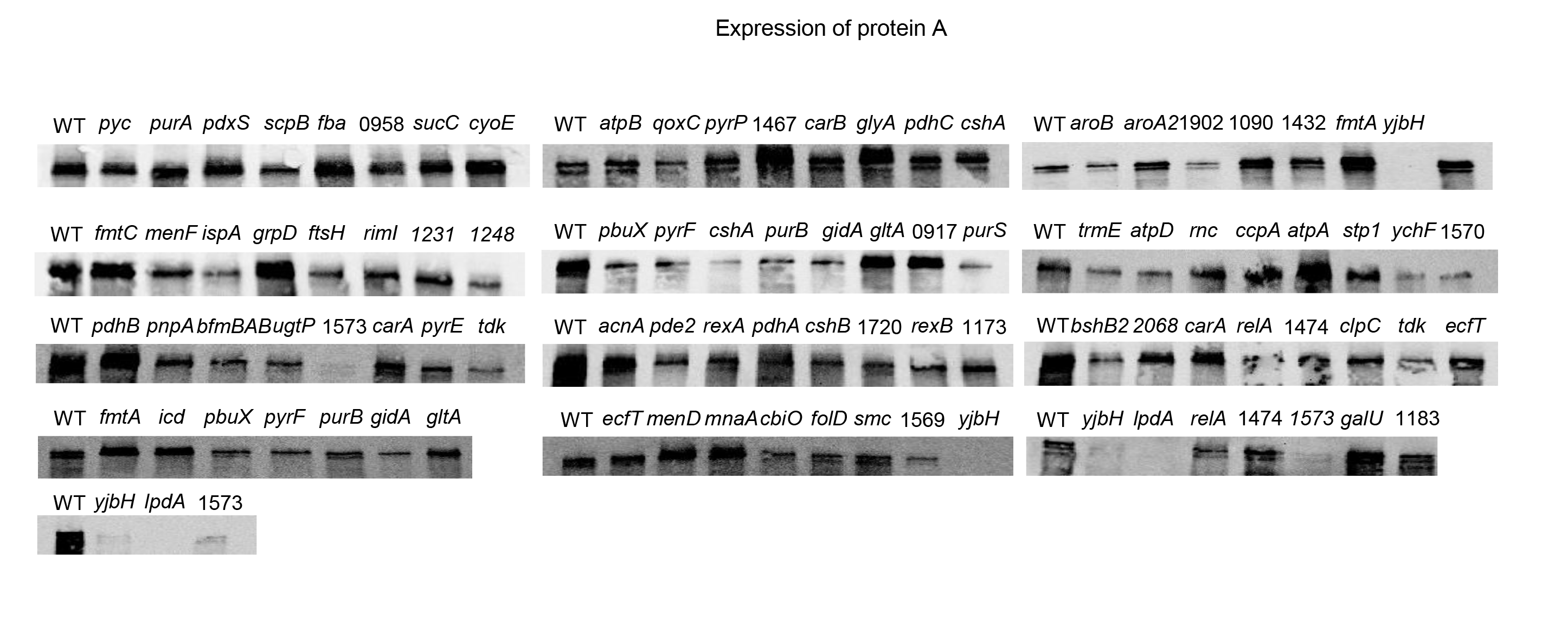

Supplement: FIG S3 [file mbio.00814-21-sf003.tif]

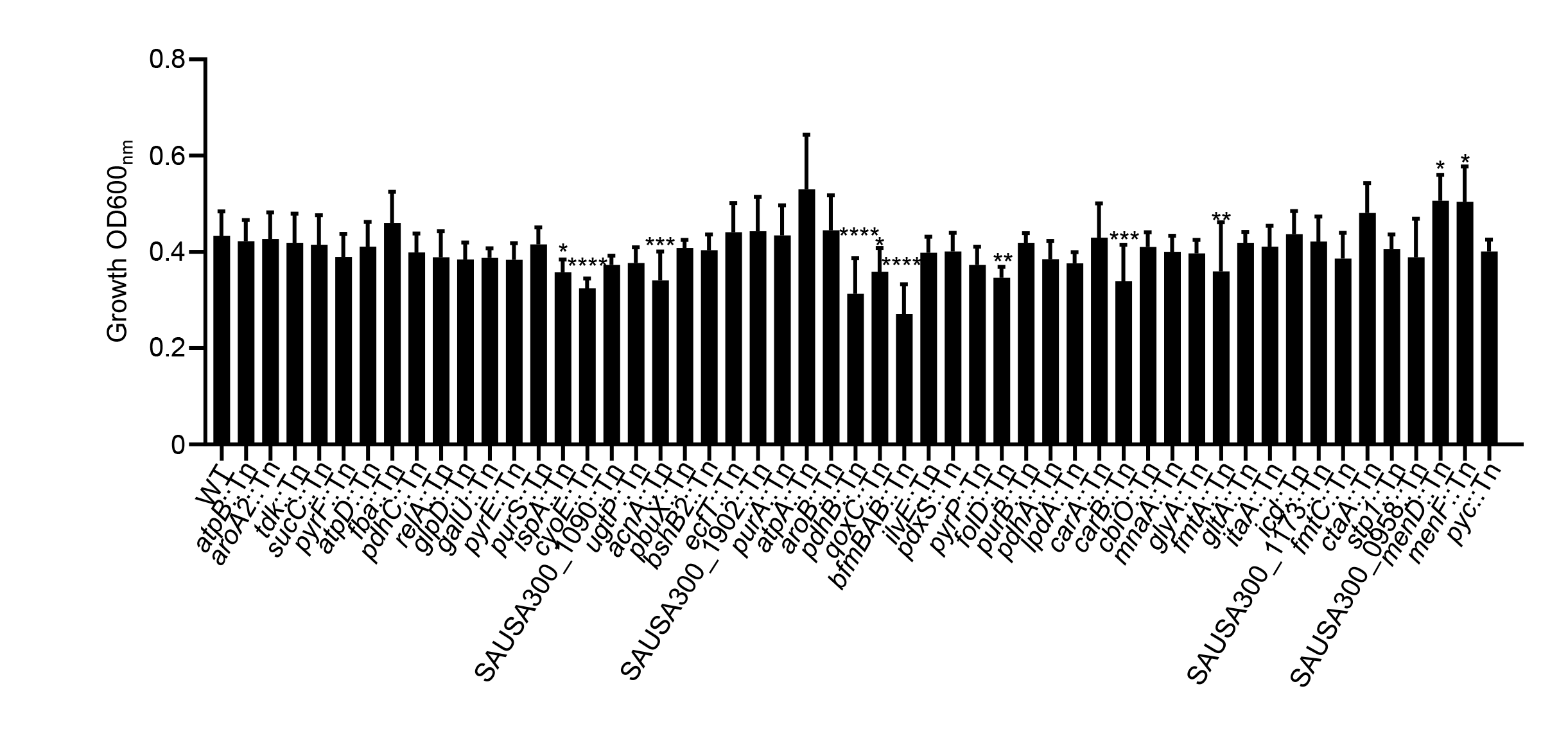

Supplement: FIG S4 [file mbio.00814-21-sf004.tif]

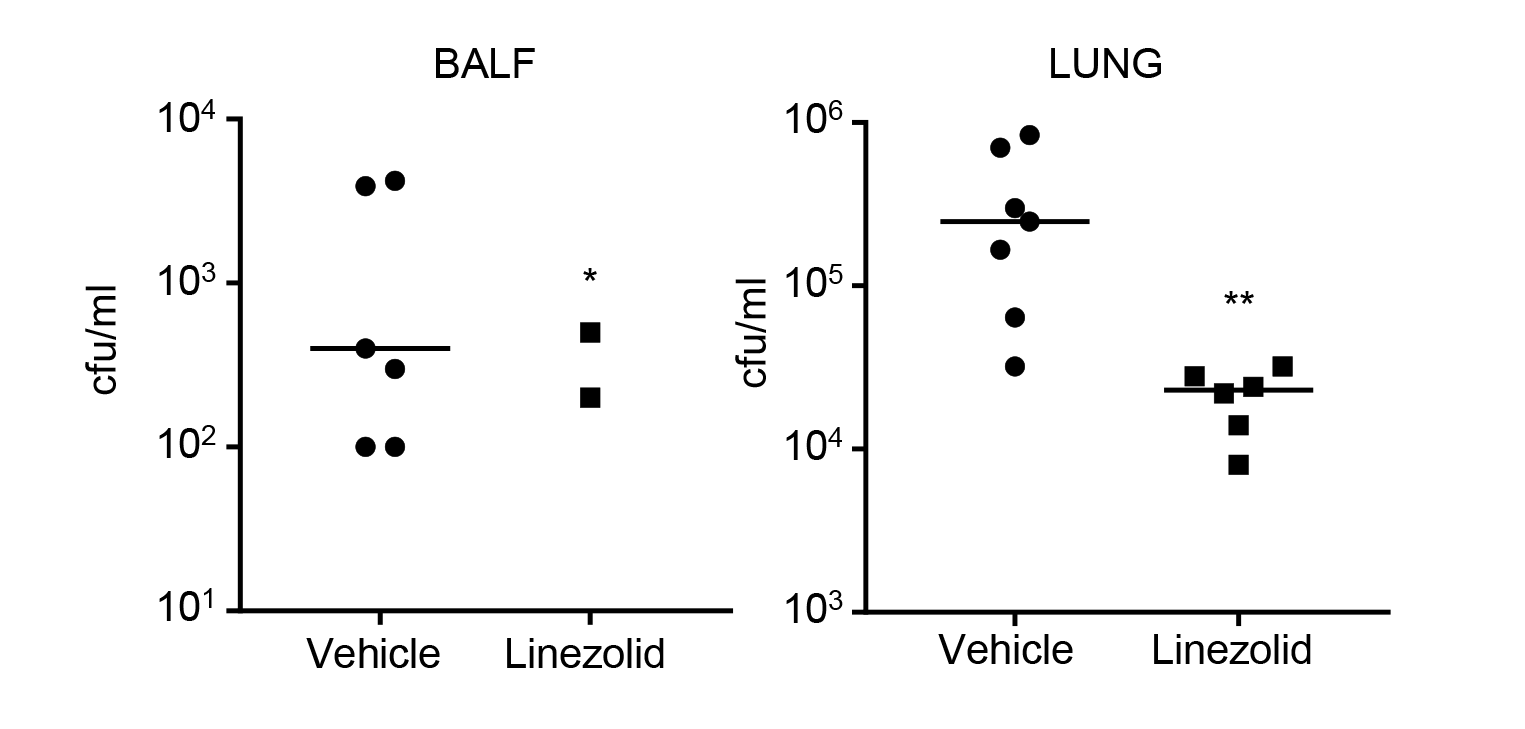

Supplement: FIG S5 [file mbio.00814-21-sf005.tif]

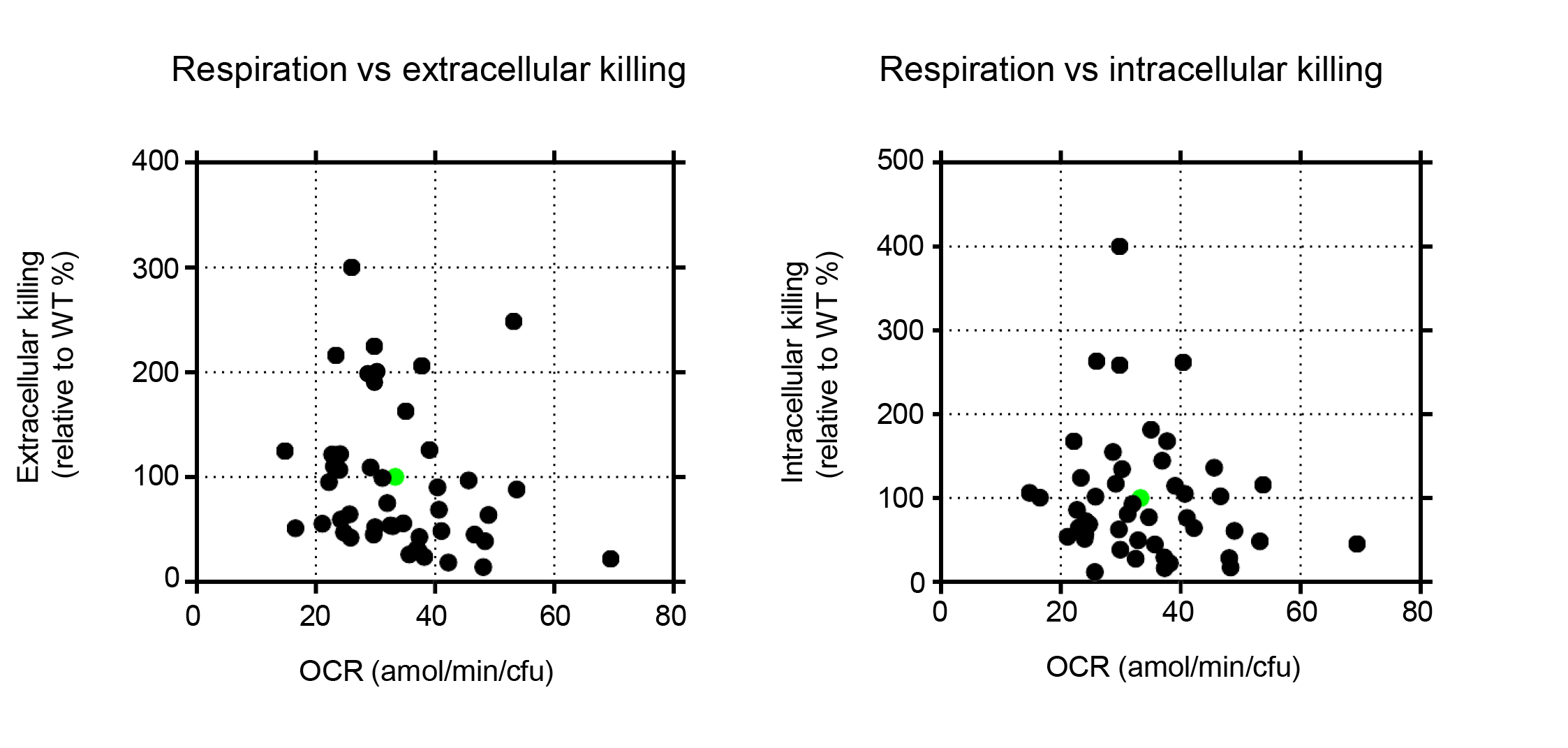

Supplement: FIG S6 [file mbio.00814-21-sf006.tif]

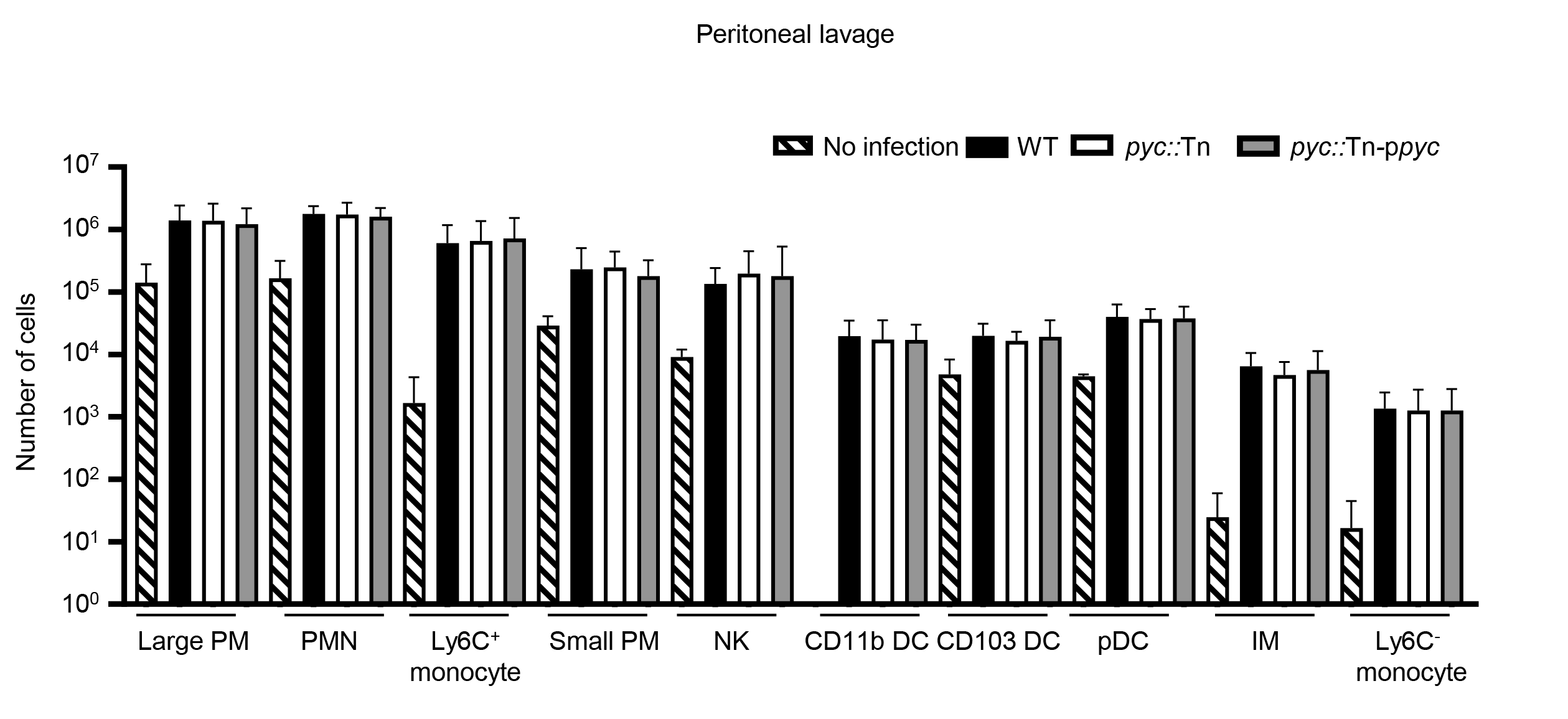

Supplement: FIG S7 [file mbio.00814-21-sf007.tif]

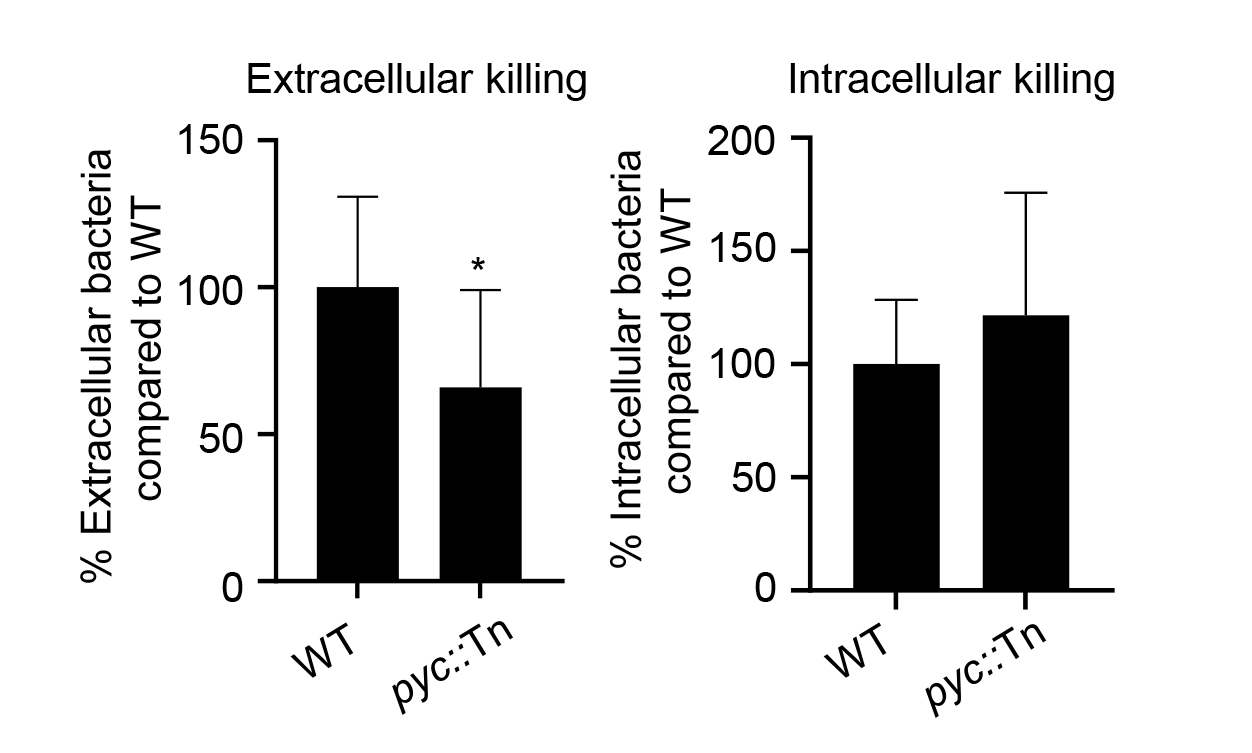

Supplement: FIG S8 [file mbio.00814-21-sf008.tif]
