# Supplementary material for: Growth and Stress Tolerance Comprise Independent Metabolic Strategies Critical for Staphylococcus aureus Infection
Source: mBio. 2021 Jun 8;12(3):e00814-21. doi: 10.1128/mBio.00814-21 (PMC8262855; doi:10.1128/mBio.00814-21)
Supplement: TABLE S1 [file mbio.00814-21-st001.docx]

Supplementary Table 1. Primers for PCR and qRT-PCR used in this study

| **Target** | **Forward primers** | **Reverse primers** | **Application** |
| --- | --- | --- | --- |
| SAUSA300_0903 | CGATCCATTTAGCTCCGATTG | CCTCGTTACATTTATGGTGTACG | PCR |
| SAUSA300_0917 | CGGTCATTCATCACAACCAC | ACGTTTTGACGATGACGAAGA |  |
| SAUSA300_1902 | GACTAACATGGAGGGGTAAAAG | ACACCTTCAGATGCTACACGA |  |
| SAUSA300_0996 | TGGTGCAACTGGCCAAAATG | GCTACAGCTTTTAATGACTGAATC |  |
| SAUSA300_0993 | TGGGAAAGGTATGGTGAATTGA | CATTGTCATTTGTGCCATGGC |  |
| SAUSA300_1138 | ACCTAAGTAACAGGAGGATGGA | GGGCAGTAGACCCTGTAATACC |  |
| SAUSA300_1889 | GTTCTGTTTTTGAAAATTCGAGT | TCATCTAACGCTGCACCTCG |  |
| SAUSA300_2067 | AAAAGATTGGTGGCGAACGT | CCGTCATTTTAGTTACAGGCGT |  |
| SAUSA300_1231 | ACATAGTCATCCCTCCACAA | TCCAATCATTGAATTTCACCCC |  |
| pLL39 &  SAUSA300_0320 | GCACATAATTGCTCACAGCCA | GCTGATCTAACAATCCAATCCA |  |
| SAUSA300_1014 | TCTAATCGAATTCGAGCTCGGT  ACCCATTACGAGATTACTCATG  ATTATCAAATTTTATAAACAAAAC | GCAGGTCGACTCTAGAG  GATCCCCAAAAGCAAAC  CGTAATTTTAATGGCAC | Complement |
| SAUSA300_1255 | GGCAATTCATACTAGTGCTTG | CTGGGCGTTTCAACCTACGT | qRT-PCR |
| SAUSA300_1014 | CAGGTGCCGATTTATTTGGTG | TCAGCACCTTGGAAACCATC |  |
| SAUSA300_2068 | AAATAGGGACTGTCGGTTCG | AACTCCCTCCAGCATGTACA |  |
| SAUSA300_1650 | CAGTGGGTGAAGCGGAATCA | CACTTGTAGATGACGCGTTCG |  |
| SAUSA300_1720 | GCATGGAGAGATGCATCTGC | GCAGCTTTTAAGAAATCATCCG |  |
| SAUSA300_2071 | GCCGAAAGCGATGATTATGAAG | CCGATATCTACGATTGTTGCATC |  |
